# Perceived utility and feasibility of pathogen genomics for public health practice: a survey among public health professionals working in the field of infectious diseases, Belgium, 2019

**DOI:** 10.1186/s12889-020-09428-4

**Published:** 2020-08-31

**Authors:** N. Van Goethem, M. J. Struelens, S. C. J. De Keersmaecker, N. H. C. Roosens, A. Robert, S. Quoilin, H. Van Oyen, B. Devleesschauwer

**Affiliations:** 1 Scientific Directorate of Epidemiology and public health, Sciensano, J. Wytsmanstraat 14, 1050 Brussels, Belgium; 2 Department of Epidemiology and Biostatistics, Institut de recherche expérimentale et clinique, Faculty of Public Health, Université catholique de Louvain, Clos Chapelle-aux-champs 30, 1200 Woluwe-Saint-Lambert, Belgium; 3 Surveillance Section, European Centre for Disease Prevention and Control, Gustav den III:s Boulevard, 169 73 Solna, Stockholm, Sweden; 4 Faculté de Médecine, Université libre de Bruxelles, 808 route de Lennik, 1070 Brussels, Belgium; 5 Transversal activities in Applied Genomics, Sciensano, J. Wytsmanstraat 14, 1050 Brussels, Belgium; 6 Department of Public Health and Primary Care, Faculty of Medicine, Ghent University, De Pintelaan 185, 9000 Ghent, Belgium; 7 Department of Veterinary Public Health and Food Safety, Faculty of Veterinary Medicine, Ghent University, Salisburylaan 133, 9820 Merelbeke, Belgium

**Keywords:** Public health practice, next-generation sequencing, pathogen genomics, whole-genome sequencing, survey

## Abstract

**Background:**

Pathogen genomics is increasingly being translated from the research setting into the activities of public health professionals operating at different levels. This survey aims to appraise the literacy level and gather the opinions of public health experts and allied professionals working in the field of infectious diseases in Belgium concerning the implementation of next-generation sequencing (NGS) in public health practice.

**Methods:**

In May 2019, Belgian public health and healthcare professionals were invited to complete an online survey containing eight main topics including background questions, general attitude towards pathogen genomics for public health practice and main concerns, genomic literacy, current and planned NGS activities, place of NGS in diagnostic microbiology pathways, data sharing obstacles, end-user requirements, and key drivers for the implementation of NGS. Descriptive statistics were used to report on the frequency distribution of multiple choice responses whereas thematic analysis was used to analyze free text responses. A multivariable logistic regression model was constructed to identify important predictors for a positive attitude towards the implementation of pathogen genomics in public health practice.

**Results:**

146 out of the 753 invited public health professionals completed the survey. 63% of respondents indicated that public health agencies should be using genomics to understand and control infectious diseases. Having a high level of expertise in the field of pathogen genomics was the strongest predictor of a positive attitude (OR = 4.04, 95% CI = 1.11 – 17.23). A significantly higher proportion of data providers indicated to have followed training in the field of pathogen genomics compared to data end-users (p < 0.001). Overall, 79% of participants expressed interest in receiving further training. Main concerns were related to the cost of sequencing technologies, data sharing, data integration, interdisciplinary working, and bioinformatics expertise.

**Conclusions:**

Belgian health professionals expressed favorable views about implementation of pathogen genomics in their work activities related to infectious disease surveillance and control. They expressed the need for suitable training initiatives to strengthen their competences in the field. Their perception of the utility and feasibility of pathogen genomics for public health purposes will be a key driver for its further implementation.

## Introduction

Sequence information from viruses, bacteria, and other infectious organisms can be used to identify a pathogen and its specific characteristics, and compare its genetic relatedness to other pathogens [1]. Advances in sequencing technologies, especially the shift to next-generation sequencing (NGS), have made it possible to analyze pathogen genomes in much greater detail. Compared to Sanger sequencing, NGS technologies allow a faster and cheaper way to sequence larger lengths of nucleotides. As such, NGS makes microbial pathogen whole-genome sequencing (WGS) accessible in high throughput within a matter of days [1]. During the last decade, NGS has expanded beyond the research settings and is being rapidly applied into routine practice for public health and food safety [1–6].

In public health, integrating pathogen genomics with epidemiology provides many opportunities for improving the population-level risk assessment and management of infectious diseases [1–8]. The main applications of WGS include (1) retrospective (or near real-time) comparisons of pathogens’ relatedness to test epidemiological transmission hypotheses of suspected outbreaks (i.e. outbreak investigations); (2) WGS-based prospective surveillance by monitoring of cases generating alerts when clusters of pathogens with similar genomes are identified in a limited geographical area or time period or when virulent clones emerge (outbreak detection by control-oriented surveillance); and (3) cross-sectional genomic epidemiology surveys to monitor long-term changes in epidemiology over larger geographic and population scales to inform prevention strategies (strategy-oriented surveillance) [9]. The main added value of implementing WGS during surveillance activities or outbreak investigations is inherent in the higher resolution of the WGS output itself, leading to an increased sensitivity and specificity to identify transmission clusters compared to conventional subtyping methods [6]. As such, there are numerous success stories of outbreak investigations applying WGS that were able to identify to the source of infection and implement targeted control measures to stop further spread, saving resources at the health protection and local authority level [10–17]. Other concrete examples of the utility of WGS for national surveillance and local infection control include the guidance of vaccination strategies [18–21] and antibiotic stewardship [22, 23]. Besides transforming the public health approach to infectious diseases monitoring, analysis of pathogen genomics can advance the accuracy of infection diagnostics and guide the treatment of individual patients [4, 24–29]. For several pathogens, NGS is able to replace current time-consuming and/or labor-intensive conventional methods with a single, all-in-one diagnostic test [30–33].

Public health professionals play a key role in protecting the population against communicable disease threats. This requires them to give effective responses in a limited time frame, supported by adequate information resulting from applying the most appropriate tools adapted to the specific public health threat scenario. Infectious disease surveillance systems build upon the cooperation between: clinicians, who are at the frontline through identification of infected patients; microbiologists, who are involved in testing specimens; molecular biologists, who study organisms at the molecular level; bioinformaticians, who develop computational approaches/algorithms to analyze genomic data; epidemiologists, who use the data to understand patterns in disease occurrence at the population level; infection control practitioners, who are responsible for local prevention and control of infectious diseases in the community; hospital hygienists, who are involved in the prevention and control of healthcare-associated infections; food safety inspectors, who monitor food products; etc. The activities of these public health experts operating at different levels in the information cycle will be impacted by the introduction of pathogen genomics as they are all connected to each other. This ranges from microbiologists adapting their laboratory workflows to epidemiologists rethinking their current data analysis approaches.

Typically, new laboratory technologies are adopted by data providers first, while data end-users might not be familiar enough with the new methods to effectively translate the output data into public health actions. Expertise with pathogen genomics and its applications for public health practice might also differ between those in charge of national surveillance of infectious diseases and those involved in local infection control and patient management, as well as between different fields (i.e. human, animal, food, and the environment) within the One Health spectrum [34, 35].

Differences in perceptions and needs between these different profiles should be taken into account before we can build a strategy that engages all the stakeholders in an effective collaboration. The key to success in translating pathogen genomics into public health practice is to demonstrate an added value by better addressing the needs and expectations of the whole range of public health experts. An effective exchange of expertise across disciplines (e.g., clinicians, microbiologists, epidemiologists, and bioinformaticians) is key for enabling the smooth implementation of NGS into routine public health activities. If such coordination of joint efforts cannot be accomplished, the technology shift, which is currently ongoing, might not realize its full potential [5, 8].

Previous surveys in the field of public health genomics focused on: human genomics [36–38]; specific aspects such as proficiency testing [39], the design of WGS clinical reports [39] or data sharing [40]; or specific target groups such as National Microbiology Focal Points [41] or food safety laboratories [42]. In this study, by organizing an online survey, we aimed to perform a wide landscape analysis of all potentially involved stakeholders in order to appraise the level of genomic literacy and to gather the opinions of public health experts and allied professionals working in the field of infectious diseases in Belgium concerning the implementation of NGS in routine public health activities, in terms of its utility, feasibility, implementation, and translation into actionable results for public health decision making.

## Methods

An electronic questionnaire survey (see Additional file 1) was developed for this study using LimeSurvey (Version 2.71.1) [43] for the collection of relevant information from public health professionals working in the field of infectious diseases in Belgium. For the purposes of this study, a ‘public health professional in the field of infectious diseases’ was defined as a person with professional expertise in the field of infectious diseases and who directly or indirectly contributes to the population-level management of infectious diseases. To provide a complete picture of all involved stakeholders, the survey aimed to reach different subgroups based on professional qualification (i.e. microbiologists, molecular biologists, bioinformaticians, epidemiologists, clinicians, clinical biologists, infection control practitioners, and hospital hygienists), employing institution (i.e. governmental, private, hospital, and university), health field (i.e. human, animal, food, and environment), expertise in pathogens (i.e. bacteria, viruses, parasites, fungi, and yeasts), and level of action (i.e. national surveillance and local infection control). To identify all actors in the field of public health activities for infectious diseases, an overview was made of existing surveillance systems (i.e. data sources) in Belgium (see Additional file 2). The set of questions was compiled based on the literature, including several review articles [1–3, 5, 7, 24–26, 44, 45]. Existing items from previous survey questionnaires [27, 36–42, 46]) were used and adapted when relevant. Most of the existing questionnaires from which some questions were adapted to be used for this survey were not validated, except for Chow-White et al. 2017 as mentioned in the respective publication [37]. The construction of the survey was discussed during several feedback rounds within a multidisciplinary team including epidemiologists, microbiologists, and molecular biologists. As a result, the survey instrument was vetted by subject matter experts. The questionnaire eventually contained eight main topics comprising background questions, general attitude towards pathogen genomics for public health practice and main concerns, genomic literacy, current and planned NGS activities, place of NGS in the diagnostic hierarchy of microbiology, data sharing obstacles, end-user requirements, and key drivers for the implementation of NGS. Based on a filter question where participants indicated their level of familiarity with pathogen genomics, the respondents were redirected to different sets of questions with different levels of technicity and detail. The filter question gave access to a reduced version of the questionnaire for those participants judging themselves as not at all familiar with pathogen genomics. The responses were mainly collected as single/multiple options from a set of pre-defined answers, but also included the optional entry of free text. These qualitative open questions were included to add context to the quantitative responses. The survey tool was pre-tested by three researchers not directly involved in the development phase to ensure the acceptability and clarity of the questionnaire.

Participants were contacted individually by an email invitation containing a personal token to complete the survey. No monetary or other incentive was offered. The participant information statement at the beginning of the survey informed the respondents about the objective and design of the study and their rights before participation to the survey, and explained that responses are anonymized and will be kept confidential. The approval from an Ethical Committee was not considered necessary due to national regulations (legislation 4 April 2014), as this study was not medical in nature and as participants were not subject to any actions and/or rules of conduct. The survey was available online during a two months’ period during which three reminders were sent to those who had not yet responded. The first invitations were sent on the 6th of May 2019 and the survey remained active until the 1st of July 2019. Participants were invited to send any questions, feedback or comments for the survey to the organizers. Only completed questionnaires were used for analysis.

Descriptive statistics were reported by analyzing categorical response frequencies. Differences in viewpoints between the stakeholders were described using subgroup analyses and compared using a Fisher’s exact test. Subgroups were compiled on the basis of the level of action (national vs. local), the position in the information cycle (data providers vs. data end-users), and the level of expertise in the field of pathogen genomics. The level of action was considered national when the main affiliation of the respondent concerned a national institute involved in national public health activities, whereas the local level included professionals who mainly operate at the community, hospital, or university level. Subgroups based on the position in the information cycle were defined as data providers, defined here as experts in wet and dry lab procedures and (potentially) generating NGS data (including microbiologists, molecular biologists, clinical biologists, and bioinformaticians), and data end-users defined here as using NGS data to improve their activities and implementing infection control measures (including epidemiologists, local infection control practitioners, hospital hygienists, and clinicians). The level of expertise was categorized as high, middle or low, and was based on respondents’ self-reported familiarity with pathogen genomics, training level, and current use of NGS. Multiple logistic regression was performed to identify predictors of a positive attitude towards the implementation of pathogen genomics from a public health perspective. Enthusiasm about public health agencies using genomics to understand and control infectious diseases was defined directly through a question with multiple options, each containing a clear statement (see Additional file 1). For the purpose of this analysis, the question asking about their enthusiasm originally consisting of multiple categories was collapsed into two levels: very enthusiastic versus all others. The following predictor variables were initially tested in the model: level of action; position in the information cycle; level of expertise; current use of NGS; institution; age group; years of professional experience; and position in their institution. Model building involved a univariate analysis to select variables to be included in the multivariable model based on a χ2-test (cut-off, P = 0.25), and variable selection from the multivariable model using backward stepwise regression based on the Akaike Information Criterion (AIC). Adjusted odds ratios (ORs) and 95% confidence intervals (CIs) were calculated. Quantitative analyses were performed using R software (R Studio version 1.0.153) [47]. Answers to open-ended survey questions were summarized and analyzed using Nvivo qualitative data analysis software (Nvivo Version 12) [48]. This was done by identifying themes (codes) within the data, which were derived both deductively and inductively. Following the thematic analysis framework, the text was compared and contrasted with the identified codes. The qualitative findings were summarized as a mind map linking the identified major and minor themes and a word cloud visualizing the word frequency from the qualitative responses. Simultaneously, quotes were selected for the sake of illustration.

## Results

### Profile of the respondents

**Fig. 1:**
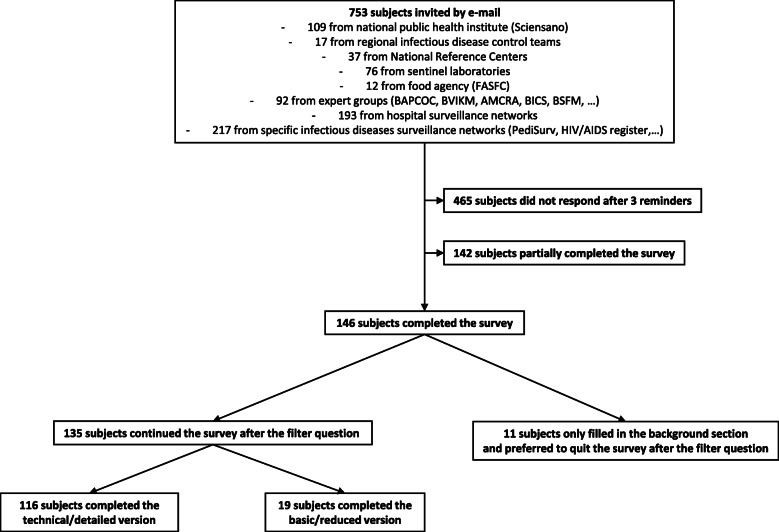
Flow chart of the participants in the survey, Belgium, 2019

Out of the 753 invited participants, 465 did not respond at all, 142 partially filled in the survey, and a total of 146 participants delivered a completed survey which represents an overall survey response rate of 19% (Fig. 1). From these, 135 participants continued after the filter question and delivered answers to all questions (116 subject were redirected to a technical version of the survey and 19 subjects to a basic version, based on the filter question). The data from the 11 participants who preferred to quit after the filter question were only used to describe the background characteristics of the study population. The 142 subjects who partially filled in the survey were dropped completely from the analysis. Full responses to all questions as they appeared in the questionnaire are provided as an appendix to this report (see Additional file 3).

Background characteristics of the 146 participants are presented in Table 1. The majority of respondents had their main affiliation in the public sector (53%), followed by hospitals (including university hospitals) (36%), private sector (8%), and university (3%). The public sector was primarily represented by Sciensano (Belgian Institute for Health), comprising 42% of all survey participants (61/146). 53% of the respondents indicated that they had more than 10 years of professional experience within the field of infectious diseases. The reported roles of respondents within their institutions included: microbiologists/molecular biologists/bioinformaticians/clinical biologists (45%); epidemiologists (21%); clinicians (13%); hospital hygienists/infection control practitioners (10%); and policy makers (3%).

**Table 1:** Demographic characteristics of the 146 public health professionals working in the field of infectious diseases who completed the survey ‘Perceived utility and feasibility of pathogen genomics for public health practice’, Belgium, 2019

Variables	Frequency	Percentage
*Primary employer*		
Public sector	77	53%
Private sector	12	8%
Hospital (including university hospital)	53	36%
University	4	3%
*Professional background*		
Epidemiologist	31	21%
Microbiologist	28	19%
National Reference Center	19	68%
Sentinel/peripheral laboratory	7	25%
Other	2	7%
Molecular biologist	16	11%
Bioinformatician	5	3%
Clinical biologist	17	12%
Clinician	19	13%
Hospital hygienist	7	5%
Infection control practitioner	7	5%
Policy maker	4	3%
Other	12	8%
*Age*		
<25	3	2%
25-34	42	29%
35-44	36	25%
45-54	32	22%
55-64	29	20%
>65	4	3%
*Years of professional experience in the field of infectious diseases*		
No experience	2	1%
<1	0	0%
1-5	32	22%
6-10	30	21%
>10	77	53%
*Position within institute/company*		
Employee	44	30%
Lower management	43	29%
Middle management	36	25%
Upper management	15	10%
Other	8	5%
*Discipline(s) (multi-discipline designation was allowed)*		
Human	119	82%
Food/feed	33	23%
Animal	26	18%
Environmental	13	9%
*Infectious diseases group(s) (multi-group designation was allowed)*		
Respiratory infections (e.g. influenza)	80	55%
Invasive bacterial diseases (e.g. *Neisseria meningitidis*)	75	51%
Vaccine-preventable diseases (e.g. measles virus)	52	36%
Consumption-related infectious diseases (e.g. *Salmonella*)	75	51%
Body-fluid related infectious diseases (e.g. HIV, hepatitis, STIs)	58	40%
Environmental-related infectious diseases (e.g. malaria)	45	31%
Healthcare-associated infections (e.g. *Clostridium difficile*, MRSA)	72	49%
Animal diseases	20	14%

**Fig. 2:**
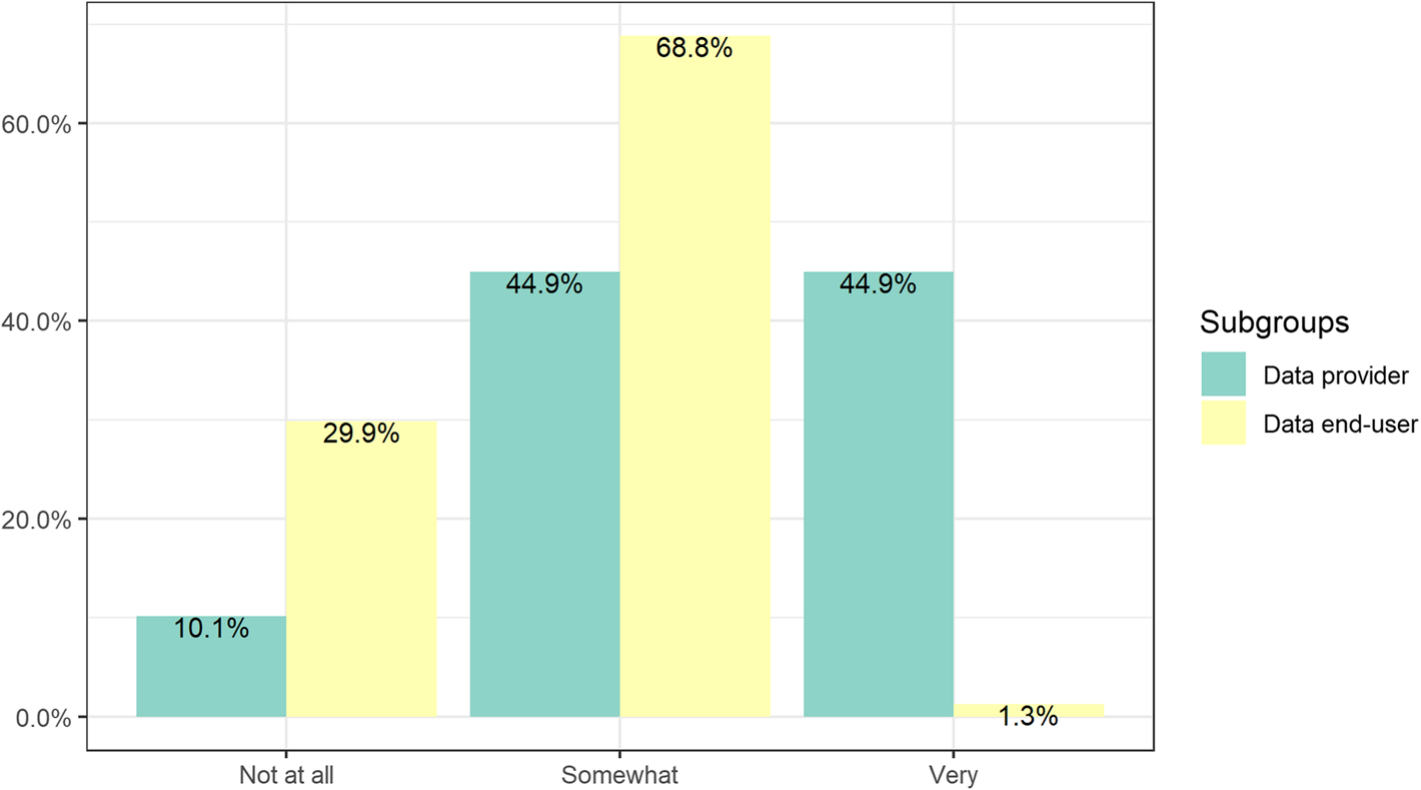
Familiarity of participants with pathogen genomics according to their position in the information cycle, Belgium, 2019. Subgroups: (i) data providers (*n*=69) including microbiologists, molecular biologists, bioinformaticians, and clinical biologists, and (ii) data end-users (*n*=77) including epidemiologists, local infection control practitioners, hospital hygienists, and clinicians. Categories: (i) ‘Very - I am involved in the generation and/or use of NGS data’, (ii) ‘Somewhat - I have a general sense of the applications of NGS’, and (iii) ‘Not at all - I don't know anything about NGS and its applications’

The 146 survey respondents were asked to describe their level of familiarity with sequencing technologies and pathogen genomics using following classification: ‘Very - I am involved in the generation and/or use of NGS data’ (22%), ‘Somewhat - I have a general sense of the applications of NGS’ (58%), or ‘Not at all - I don't know anything about NGS and its applications’ (21%). Of those participants answering ‘very familiar’, most of them (88%; 28/32) indicated that they mainly used NGS in the context of WGS. Of those ‘not at all familiar’, 11 preferred to quit the survey and 19 continued the survey to answer some general questions, leaving a total of 135 participants for the remainder of the survey (Fig. 1). Subgroup analysis showed differences in familiarity with pathogen genomics between data providers and end-users (Fig. 2). Data providers indicated significantly more frequently that they were ‘very familiar’ compared to data end-users (*p* < 0.001).

### Attitude

**Fig. 3:**
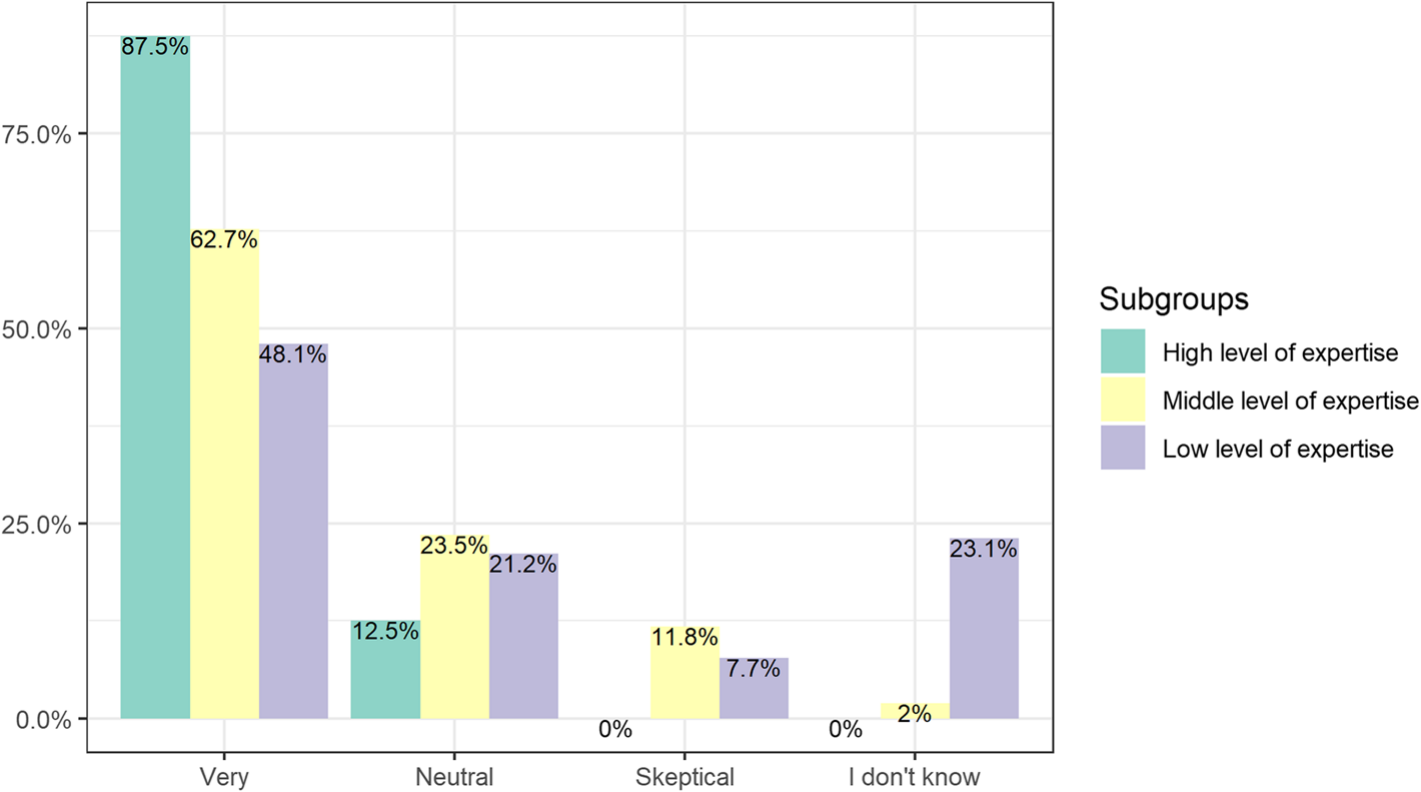
Enthusiasm of participants regarding the use of pathogen genomics for public health practice according to level of expertise in the field of pathogen genomics, Belgium, 2019. Subgroups: (i) High (*n*=32) (indicated ‘Very familiar’ and currently generating or using NGS data or continuing education in the field of genomics or professional experience in the field of genomics), (ii) Middle (*n*=51) (indicated ‘Somewhat familiar’ and having followed training in the field of genomics), and (iii) Low (*n*=52) (indicated ‘Not at all familiar’ or indicated ‘Somewhat familiar’ and having never followed training in the field of genomics). Categories: (i) ‘Very enthusiastic – we should be using genomics now’, (ii) ‘Neutral – I do not have an opinion on genomics in public health’, (iii) ‘Skeptical – genomics may be useful for research purposes, but I do not see clear applications and/or an added value for public health’, (iv) ‘It’s all a hype – genomics has not proven itself to be more useful than the conventional methods, we should not invest resources/time in genomics’, and (v) ‘I don’t know – I don’t know enough of the topic to be able to give an opinion’

The majority of respondents (63%; 85/135) indicated that they were very enthusiastic (i.e. ‘we should be using genomics now’) about public health agencies using genomics to understand and control infectious diseases, 30% (40/135) did not have an opinion or did not know enough of the topic to be able to give an opinion, and 7% (10/135) indicated that they did not see clear applications and/or an added value for public health. Subgroup analysis pointed out differences in enthusiasm according to the level of expertise in the field of pathogen genomics (Fig. 3). Important predictors, as identified by the best fitting model, of a positive attitude related to the implementation of pathogen genomics from a public health perspective were the level of expertise, the level of action, and the position in the information cycle (Table 2). Participants classified as having a high level of expertise based on their self-reported familiarity with the topic, their training level, and/or the current use of NGS were significantly more likely to be enthusiastic about the implementation of pathogen genomics in a public health context compared to their peers with a low expertise (adjusted OR = 4.04, 95% CI = 1.11 – 17.23). Further, public health professionals operating at the national level were more often ‘very enthusiastic’ about the implementation of pathogen genomics (71%) compared to those at the local level (53%). Similarly, data providers were more often ‘very enthusiastic’ (76%) compared to data end-users (50%).

**Table 2:** Determinants of public health professionals’ positive attitude towards the implementation of pathogen genomics in a public health context, Belgium, 2019

Variables^**a**^	Total	Very enthusiastic^**b**^	Crude	Adjusted^**c**^
	N	n (%)	OR (95% CI)	OR (95% CI)
Level of expertise				
Low (reference)	52	25 (48.1)	1.00	1.00
Middle	51	32 (62.7)	1.82 (0.83 – 4.04)	1.54 (0.68 – 3.52)
High	32	28 (87.5)	7.56 (2.53 – 28.27)	4.04 (1.11 – 17.23)
Level of action				
Local (reference)	57	30 (52.6)	1.00	1.00
National	78	55 (70.5)	2.15 (1.06 – 4.42)	2.07 (0.96 – 4.52)
Position in information cycle				
Data end-user (reference)	68	34 (50.0)	1.00	1.00
Data provider	67	51 (76.1)	3.19 (1.55 – 6.78)	2.09 (0.88 – 5.09)

*OR* odds ratio, *CI* confidence interval

^a^Variables were selected using backward stepwise regression based on the Akaike Information Criterion from a multivariable logistic model

^b^Participants were classified as those who were ‘very enthusiastic – we should be using genomics now’ concerning the implementation of pathogen genomics in public health practice vs. all others

^c^Odds ratios adjusted for the set of variables included in the final multivariable logistic regression model (i.e. level of expertise, level of action, and position in information cycle)

### Expected impact

A large majority of respondents considered the following public health activities as likely to be most impacted by pathogen genomics in the next five years: identifying an outbreak (clusters of related isolates) (78%; 105/135), nosocomial and food/waterborne outbreak investigations (76%; 103/135), and monitoring the spread of antimicrobial resistance (71%; 96/135). In contrast, only 44% (60/135) of respondents thought that pathogen genomics would have a major impact on making a diagnosis and selecting an appropriate treatment (individual patient management). Other public health activities that will benefit from the implementation of pathogen genomics mentioned by the participants are presented in Table 3.

**Table 3:** Illustrative quotes selected from the qualitative survey data, Belgium, 2019

Topic	Subtopic	Illustrative quotes
Public health activities, other than those provided within the survey, that will benefit from the implementation of pathogen genomics	Environmental monitoring	*“drinking water quality”* *“air quality, home environmental quality”*
	Metagenomics	*“metagenomics for patients with no identified cause of illness using conventional methods”* *“identification and characterization of new strains”* *“insights in dysbiosis”* *“microbiome analysis”*
	Other	*“discovery of a causal relation between a pathogen and a clinical disease (e.g. cancer)”* *“vaccine development”* *“phage therapy”* *“early diagnostics of diseases due to slow growing pathogens”* *“international tracking”* *“monitoring of antiviral resistance”*
Concerns, other than those provided within the survey, related to the implementation of pathogen genomics for public health practice	Contextual data	*“harmonization of epidemiological data – most of the epidemiological data is very ‘messy’ or inconsistent, which makes systematic integration and surveillance unfeasible”* *“data collection is already limited so newer technologies will not automatically improve this process but be redundant if the basics are not met”*
	Interpretation and education	*“how to interpret the result at clinical level”* *“[…] they need to have a basic understanding (education) it order to understand and see cost/benefit of the whole picture”* *“appropriate training of personnel for execution and interpretation”* *“interpretation across sectors”* *“multidisciplinary knowledge”*
	Ethics	*“[…] healthcare workers integrity concerns”* *“in the HIV field, the phylogenetic analyses of virus permit to have an hindsight in paths of transmission – it is a very tricky topic in ethical and potentially legal aspects”*
	Other	*“does the identification prove that the pathogen poses a risk?”* *“the fear that some actors in the field will try to abuse their power and monopolize this new technology – to be really valuable to patient management and public health it is required to offer access to all laboratories”* *“high inter-laboratory variability”* *“[…] standardization and facilities for data sharing need to be improved”* *“the perceived utility and feasibility of pathogen genomics by public health practitioners is the biggest bottleneck of all – all the other concerns listed above can be tackled given the drive within the field to solve them in the first place”*
Reasons, other than those provided within the survey, for not taking a training/course in the field of pathogen genomics		*“lack of training adapted to public health needs”* *“not applicable for a clinician”* *“not my priority”* *“not relevant for my practice”*
Key drivers for implementation of pathogen genomics		*“depends on the evolution in phenotypic typing”* *“[…] the main driver the pressure by ECDC rather than a real need for public health […] the first and main driver should be clinical significance: improve quality of care for the patient”* *“for bacteria, NGS will never fully replace classical methods for resistance testing, but would offer important complementary data”* *“cost-effectiveness (e.g. replacing multiple tests): not particularly true for viruses, but obvious for bacteria”*
Laboratory workflow integration	Centralization	*“[…] should be overall coordinated and controlled by the federal public health authority”* *“[…] in any scenario it will be important that sequence data are brought together in one databank for surveillance purposes”*
Data sharing	Organizational aspects	*“no central BE or EU organization”* *“no central database, no clear guidelines on how and what to share”* *“the bureaucracy involved in the transmission of data”* *“the structure of public health in Belgium will not help sharing data”* *“the required technical infrastructure”*
	Priority to publication	*“it is really a pity that priority to publication is an obstacle in the scientific world as it functions now”*
One Health		*“A better collaboration between the veterinary and human side might increase the use of NGS on the veterinary side”* *“Monitoring the emergence and spread of zoonotic pathogens has been impacted negatively, by the introduction of WGS at the human side only”*

### Concerns

The most frequent concerns among participants being ‘very’ or ‘somewhat’ familiar with NGS technologies and pathogen genomics (*n*=116) regarding feasibility of its routine use for public health purposes, were the cost of sequencing technologies and the existing barriers to timely and open sharing of pathogen sequence data and accompanying metadata (Table 4). All participants exclusively working with respiratory infections (e.g. influenza) and/or vaccine-preventable diseases (e.g. measles) (*n*=7) were very concerned about the cost, whereas this was only true for 38% of participants exclusively working with invasive bacterial diseases (e.g. *Neisseria meningitidis*), food- and waterborne diseases (e.g. *Salmonella*), and/or healthcare-associated infections (e.g. *Clostridium difficile*) (*n*=21). Further, other concerns shared by a large proportion of the participants were interdisciplinary cooperation, integration of pathogen sequence data with contextual data, access to bioinformatics expertise, and availability of typing schemes and databases. Participants indicating to be ‘not at all’ familiar with pathogen genomics were mainly concerned about the cost of the sequencing technologies (see full responses in Additional file 3). Other concerns provided by the participants as free text are presented in Table 3.

**Table 4:** Concerns among participants being ‘very’ or ‘somewhat’ familiar with pathogen genomics expressed as the percentage being ‘very’ or ‘somewhat’ concerned, Belgium, 2019. Subgroups: (i) data providers (*n*=62) including microbiologists, molecular biologists, bioinformaticians, and clinical biologists, and (ii) data end-users (*n*=54) including epidemiologists, local infection control practitioners, hospital hygienists, and clinicians

	Overall (*n*=116)	Data providers (*n*=62)	Data end-users (*n*=54)
Quality of the pathogen sequence data (validation and accreditation of both wet and dry lab protocols)	63%	73%	52%
Timeliness of the pathogen sequence data (turn-around time)	67%	76%	57%
Integration of pathogen sequence data with other types of data (e.g. clinical and epidemiological data)	70%	61%	79%
Linking pathogen sequence data from different sources (human/food/animal/environment)	59%	55%	64%
Translation of pathogen sequence data into public health action (usefulness)	68%	64%	71%
Interdisciplinary working/coordination between epidemiologists, microbiologists, bioinformaticians, etc.	75%	79%	70%
Cost of sequencing technologies	86%	87%	85%
Expertise and availability of personnel to be able to generate pathogen sequence data (wet lab)	60%	64%	55%
Expertise and availability of personnel to be able to analyze pathogen sequence data (bioinformatics)	75%	82%	67%
Timely and open sharing of pathogen sequence data and accompanying metadata	81%	89%	72%
Infrastructure (sequencers, high-performance computing, data storage, etc.)	67%	74%	59%
Availability of WGS typing schemes and reference databases (e.g. for antimicrobial resistance)	73%	79%	67%
Ethical and legal issues (e.g. patient privacy)	54%	56%	52%

### Genomic literacy

**Fig. 4:**
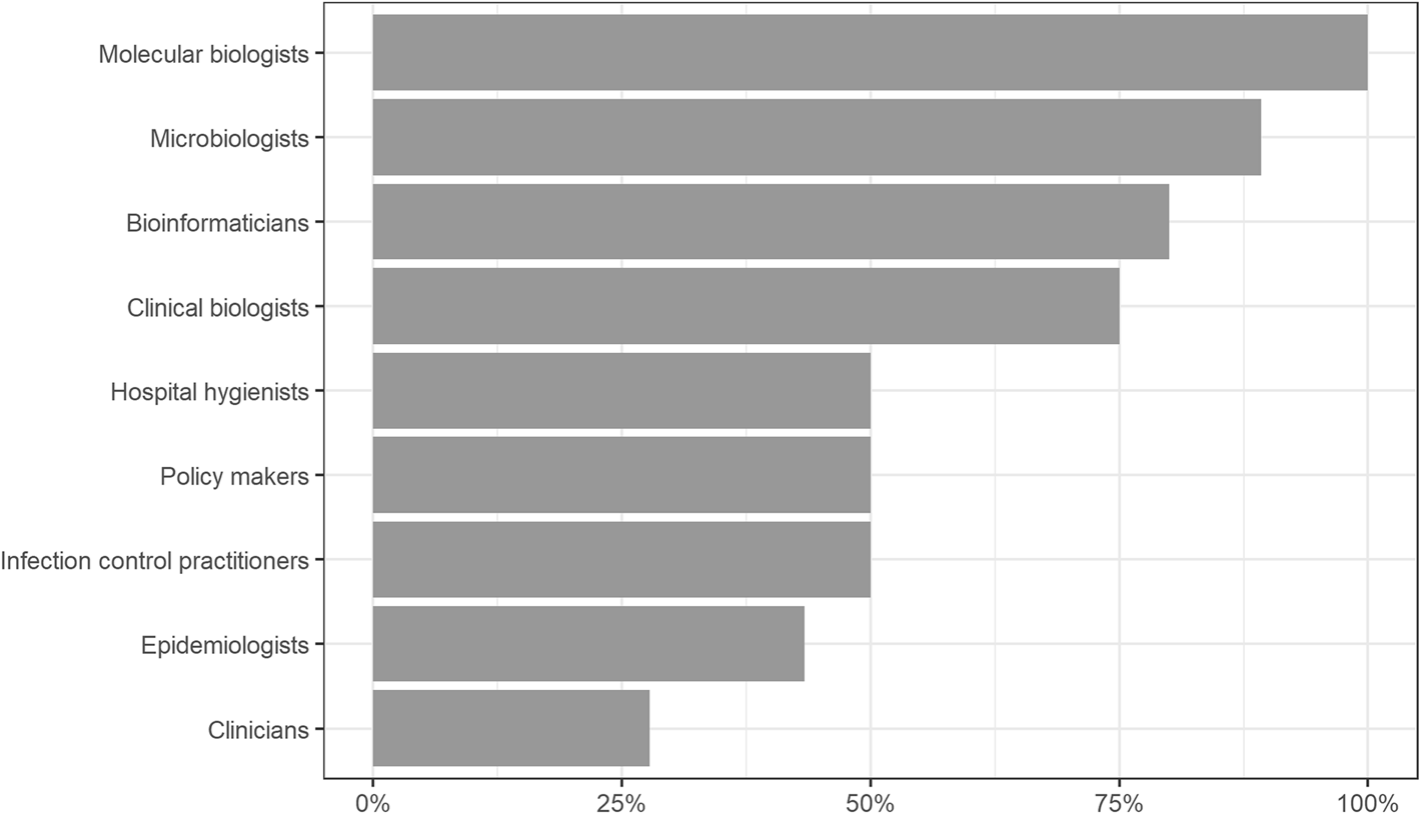
Percentage of respondents who had received training in pathogen genomics by professional category, Belgium, 2019

Two-thirds of the participants (88/135) indicated that they had followed training in the fields of genomics/genetics/molecular biology/bioinformatics. There were marked differences by position in the information cycle: 56% (38/68) of data end-users indicated that they had never followed any training in the field, whereas this was stated by only 13% (9/67) of data providers (p < 0.001). Further breakdown of training experience by professional category is shown in Fig. 4.

The main reasons for not taking a training/course in this field (yet) were the lack of available and/or suitable trainings (40%; 19/47) and the lack of time (30%; 14/47). Other reasons indicated as free text are presented in Table 3. The vast majority of participants (79%, 106/135) indicated that they felt the need and/or would be interested in following (additional) courses/training/workshops covering a topic related to pathogen genomics.

### Current and planned NGS activities

**Fig. 5:**
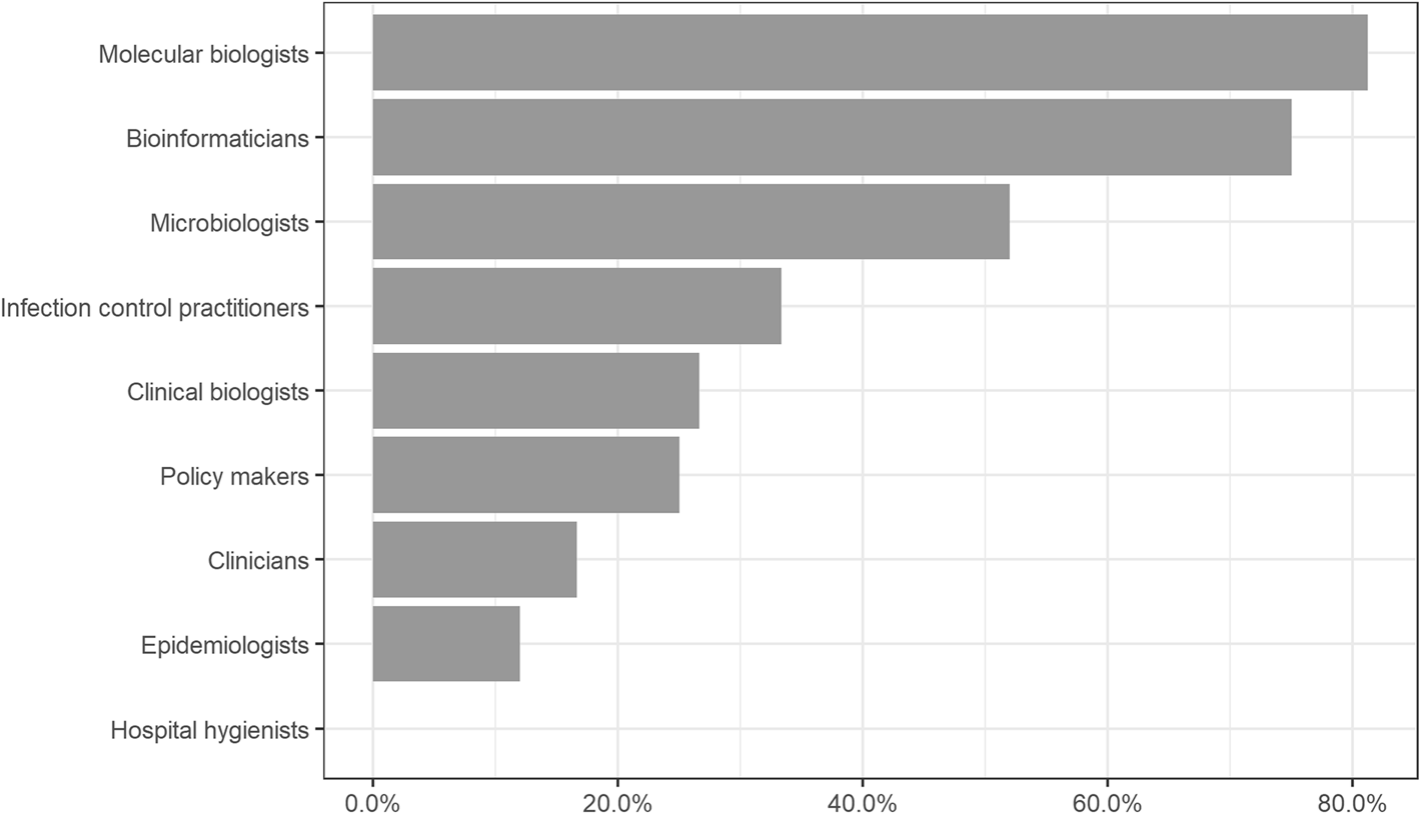
Percentage of NGS current users by professional category, Belgium, 2019

Overall, 36% (42/116) participants being ‘very’ or ‘somewhat’ familiar with NGS technologies and pathogen genomics indicated that they are currently using or generating NGS data for at least one pathogen. Differences between professional groups are presented in Fig. 5. Among the microbiologists, those from a National Reference Centre (NRC) were more likely to be currently using NGS (12/18, i.e. 67%) compared to those from other laboratories (1/7, i.e. 14%), however this difference was not significant (p=0.71). From the public health professionals exclusively involved in human infectious disease activities, 30% (21/70) were currently using NGS technologies, whereas this was the case for 44% (11/25) of those exclusively involved in the food, animal or environmental sector (p=0.23).

Looking forward, 44% (51/116) of participants indicated that they were planning to use or generate NGS data for any (additional) pathogen(s) within three years. Details on the specified pathogens can be found in the appendix (see Additional file 3). Reasons provided by participants indicating that they did not plan to implement pathogen genomics were mainly related to the cost and the lack of expertise.

### Key drivers for implementation

**Fig. 6:**
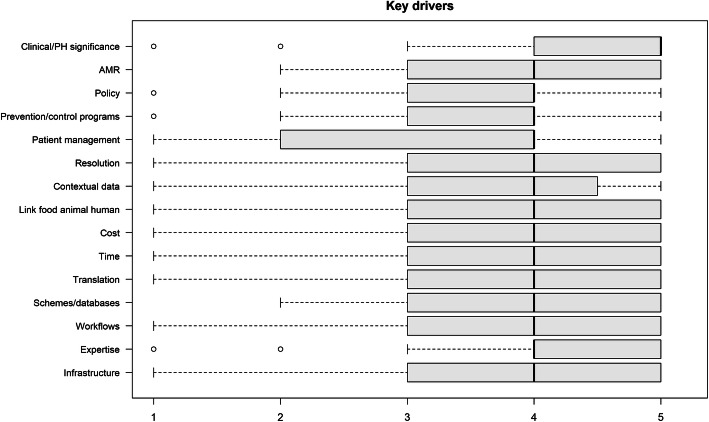
Boxplots of the scores of different key drivers for implementation of pathogen genomics rated by 116 participants, Belgium, 2019. Criteria could be assigned a score from 1 to 5, or participants could indicate the ‘I don’t know’ option. The boxplots show the median score and the interquartile range (grey boxes). The following criteria were included (top to bottom): clinical and/or public health significance, priority with respect to preventing the spread of antimicrobial resistance, local/national/international policy surveillance priorities or obligations, importance of prevention and control programs (e.g. vaccination), utility of WGS for diagnostics and/or treatment decisions (individual patient care), utility of increased resolution to infer relatedness that would not be obtained via conventional methods, availability of high-quality/complete/standardized epidemiological and/or clinical data to provide context to the WGS results, possibility to link genomic data from different sources (food-animal-human-environment), cost-effectiveness (e.g. replacing multiple tests), time-saving compared to conventional testing methods, impact on outcomes for patients and populations (translation into actionable results), availability of WGS typing schemes and reference databases (e.g. for antimicrobial resistance), availability of validated (quality-controlled) WGS workflows (both wet and dry laboratory), availability of expertise to generate, analyze and interpret WGS data, and availability of the appropriate infrastructure (sequence technology, high-performance computing, data storage, etc.).

Participants being ‘very’ or ‘somewhat’ familiar with NGS technologies and pathogen genomics (*n*=116) were asked to assign a score from 1 to 5 to the different criteria based on their increasing relative importance to decide whether or not NGS should be implemented for a particular pathogen (Fig. 6). Clinical and/or public health significance of the pathogen were scored as the most important drivers. The different subgroups scored the different criteria similarly (see Additional file 3). Comments provided by the participants to provide context to their scores are presented in Table 3.

### Laboratory workflow integration

**Fig. 7:**
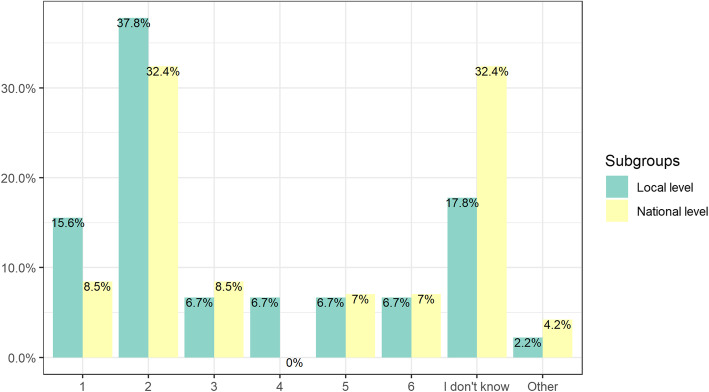
Percentage of participants (*n*=116) selecting a particular WGS provision model by level of action, Belgium, 2019. 1 = Centralization of sequencing and bioinformatics at one central sequencing center, 2 = Centralization of sequencing and bioinformatics at National Reference Centers (which are organized per pathogen or group of pathogens), 3 = Decentralized point-of-care sequencing (at frontline laboratories), but centralization of bioinformatics (mixed model), 4 = Decentralization of sequencing and bioinformatics, but mandatory submission of isolates to a national repository, 5 = Decentralization of sequencing and bioinformatics, but mandatory submission of raw sequence data to a national repository, 6 = Decentralization of sequencing and bioinformatics, but mandatory submission of bioinformatics output to a national repository

Centralization of sequencing and bioinformatics at NRCs organized per pathogen or per group of pathogens was most often (34%; 40/116) selected by respondents being ‘very’ or ‘somewhat’ familiar with pathogen genomics as the preferred WGS provision model in the Belgian context. Excluding participants working at NRCs slightly lowered this proportion to 29 out of 98 (i.e., 30%). There were no marked differences according to the level of action of the participants (Fig. 7). Illustrative quotes for the need for centralization are presented in Table 3.

### Data sharing

A majority (59%; 68/116) of the participants being ‘very’ or ‘somewhat’ familiar with pathogen genomics considered technical barriers (lack of data standardization, poor data quality, missing metadata, etc.) as a major obstacle for sharing pathogen sequence data and associated metadata, whereas only 38% (44/116) cited ethical issues and concerns, 35% (41/116) political sensitivities, and 27% (31/116) priority to scientific publication. The proportion of participants indicating priority to scientific publication as a major obstacle for data sharing did not significantly differ between those primarily affiliated to a university (including university hospitals) compared to the other participants. Concerns about organizational aspects are presented in Table 3.

### Qualitative analysis results

**Fig. 8:**
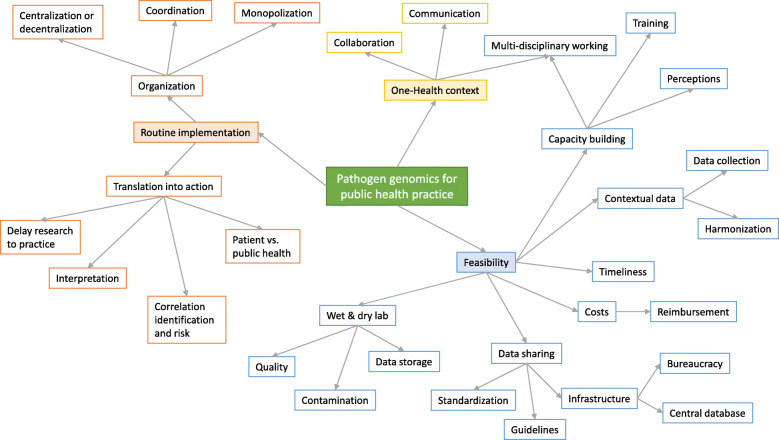
Mind map linking the major and minor themes identified in the qualitative responses, Belgium, 2019. Codes were identified within the data deductively (i.e. themes that are expected and have been chosen in advance) and inductively (i.e. themes that are derived through analysis). During the thematic analysis the qualitative data from the survey was compared and contrasted with the identified codes. As such, the derived codes were assigned to the relevant text. Next, the codes (plain boxes) were merged into categories (colored boxes). The following categories were identified: routine implementation (orange), One-Health context (yellow), and feasibility (blue)

Major themes identified within the qualitative data are utility (applications), feasibility (including capacity building, multi-disciplinary working, contextual data, costs, data sharing, ethics, timeliness, wet and dry lab), One Health context, and routine implementation (including organization and translation into action). A mind map linking the identified major and minor themes is presented in Fig. 8. A full list of identified themes and the coded text is available in the appendix (see Additional file 4), as well as a word cloud constructed based on the free text responses (see Additional file 5).

## Discussion

This survey sought the opinion of Belgian public health professionals working in the field of infectious diseases concerning the implementation of pathogen genomics in public health activities. To successfully translate pathogen genomics into public health practice, the needs and expectations of the different stakeholders should be taken into account. Other questionnaire surveys related to knowledge and attitudes towards public health genomics in specific health expert categories have been published [27, 36–39, 41, 42, 46, 49]. However, to the best of our knowledge this survey is the first that aimed to perform a wide landscape analysis of all potentially involved stakeholders. Therefore, a strength of the current study is that it took into account a wide range of stakeholders with diverse backgrounds (epidemiologists, microbiologists, bioinformaticians, clinicians, infection control practitioners, etc.), health domains (human, food, environmental, etc.), pathogen expertise (bacteria, viruses, parasites, fungi, etc.), activity sectors (public, private, university, hospital, etc.), work positions (employee and lower/middle/high management), and degree of familiarity with genomics. Besides seeking the general attitude of the participants towards the implementation of pathogen genomics in their professional activities and investigating the current and future use, this explorative study was able to touch upon multiple key topics, such as genomic literacy, data sharing obstacles, place of NGS in the diagnostic hierarchy of microbiology, and end-user requirements.

Familiarity with sequencing technologies and pathogen genomics varied between the different professional groups, with data providers being more familiar than data end-users. As shown before, one of the largest barriers to acceptability from the public health unit is the capacity to understand and use the data [50]. Possibly, there is a positive association between genomic literacy and (critically) predicting the added value in public health. Having a high level of expertise, was the strongest predictor for a positive attitude, as was also shown in other surveys [51–53]. Epidemiologists and infection control practitioners should be informed about the benefits and limitations of NGS technologies in order to contribute in identifying tangible field application in public health, allowing the use of WGS output to appropriately guide public health actions [38, 51]. Another important challenge related to the interpretation of WGS data is the capacity to interpret signals, and thereby separating noise from public health events that require specific actions. Consequently, integrating genomics into infection control and surveillance is critically linked to human resource development [8, 24]. In the survey, the main reasons stated for not training in the field of genomics were lack of time or access to suitable trainings *“…adapted to public health needs*”. However, the participants of this survey generally expressed a positive attitude towards following (additional) training courses, or workshops in pathogen genomics. Educational workshops should be applied to a public health context and bring together the expertise of microbiologists, molecular experts, bioinformaticians, epidemiologists, infection control practitioners, and clinicians. The development of a new discipline called ‘genomic epidemiology’ integrating information on epidemiological and pathogen sequence characteristics by public health microbiologists, epidemiologists, and risk managers was recommended in the expert opinion on WGS for public health surveillance by the European Centre for Disease Prevention and Control (ECDC) in 2016 [8]. ECDC has initiated public health genomics training workshops that bring together experts with epidemiology, microbiology and pathogen genomics backgrounds from European Union (EU) member states with interest in implementing the technology in surveillance and outbreak investigations. Besides, the zoonotic origin of many clinically relevant pathogens and antimicrobial resistance determinants stresses the importance of a cross-sectoral One Health approach. The implementation of WGS should be synchronized and integrated between the human health and veterinary sectors [9] allowing a better monitoring of the emergence and spread of zoonotic pathogens and antimicrobial resistance-related threats.

Lack of financial resources was often indicated as a principal reason for not using or planning to use WGS by the respondents of this survey, which was also reported by the European surveys conducted by ECDC [41] and the European Food Safety Authority (EFSA) [42]. Operational costs will be influenced by the processes used in current laboratory practice and differs between viruses and bacteria. Whereas drug susceptibility testing and epidemiological typing are commonly performed for bacteria, this is often not the case for viruses detected in the routine laboratory [54]. Therefore, cost-effectiveness of NGS for many bacteria potentially follows from the replacement of conventional characterization methods, whereas for viruses NGS is considered as a tool providing additional complementary information without replacement of the existing methods. Further, an important consideration is the added value of NGS for routine diagnostics. As long as NGS is more expensive than the conventional methods and when there is no direct benefit for the individual patient, it will not be used in routine. Then the fields of application for surveillance purposes should be clearly defined to be able to justify the additional financial resources needed to perform WGS beside the diagnostic activities.

To translate pathogen sequence data into truly useful and actionable information, it needs to be integrated with other types of information (i.e. clinical and epidemiological data). In Belgium, most data end-users were concerned about the challenges encountered with the integration of pathogen sequence data with clinical and epidemiological data. Indeed, the public health usability of any kind of lab results, including WGS data, is highly dependent on the cross-linkage with contextual epidemiological and clinical information [8, 55, 56]. Data integration is often hampered by the incomplete and/or unstandardized nature of the contextual data [57]. The ongoing digitalization of health data such as laboratory and clinical records may represent an opportunity to review and upgrade traditional data collection processes for communicable disease surveillance.

According to World Health Organization’s (WHO) 2016 guidance on managing ethical issues in outbreaks [58], rapid data sharing is crucial during an unfolding health emergency. This suggests that pathogen sequence data should be rapidly and openly shared at the start of an outbreak, in many cases before scientific publication. However, many barriers for data sharing remain including authorship/attribution for publications, results dissemination, ethical considerations, data ownership, database access agreements, etc. [59]. In our survey, practical barriers (lack of data standardization, poor data quality, missing metadata, etc.) seemed to be the major obstacles in Belgium for sharing pathogen sequence data and associated metadata for public health purposes. Participants mainly mentioned the lack of a central database and clear guidelines. This reflects a lack of information on the effective data sharing through EU-wide genomic surveillance and cross-border outbreak analysis systems managed by ECDC and EFSA in support of the member states [9, 60, 61]. Finally, 27% of participants considered the priority to publication as a major bottleneck for sharing pathogen sequence data. Publication priority is linked to the importance of guaranteeing reputational returns to research efforts [40, 62]. The challenge here is to find a balanced arrangement that allows data sharing in real time and the acknowledgement of research work by giving to researchers who have been involved in data generation the possibility to use and publish their own results in priority. As the use of NGS shifts from research to routine laboratory practices, this data sharing barrier will slowly be alleviated.

Regarding expertise and availability of personnel, wet and dry lab experts were more concerned about the analysis of pathogen sequence data than the sequencing itself. As was mentioned in a review article of Aarestrup et al. and documented in a recent European survey by Revez *et al.*, the most important limiting factor in many countries is the lack of access to bioinformatics expertise, especially when used as part of frontline diagnostics [44] or national public health reference laboratory service [41].

Another point of discussion is the potential impact of NGS on the diagnostic microbiology pathway. Traditionally, frontline clinical laboratories perform standard identification, antimicrobial susceptible testing and occasionally typing. Isolates may then be referred to reference laboratories based on the need (e.g. diagnostic confirmation) or for surveillance purposes. These reference laboratories perform confirmation testing and advanced characterization. NGS was first implemented at the level of academic or reference laboratories, because of the need for investments, operational costs, and requirements for expertise [24] while having limited added value for individual patient care. Samples must be multiplexed (batching) for cost-effectiveness, which is easier to achieve in large reference laboratories with high volume of sample throughput [26, 45]. However, processing delays may be present when samples are shipped to a reference center. These processing delays may result in longer turnaround times rendering this centralized approach inappropriate to support a fast response when needed. The reduced costs of sequencing facilitated the introduction of NGS technology to frontline clinical laboratories. This shift towards a decentralized use may reduce turnaround times, empower hospital-based microbiology, and strengthen local infection control efforts [24]. This decentralized capacity will allow the inclusion of these data in the surveillance network coordinated by the epidemiologists what will compensate the reduced referral of isolates to reference centers. Consequently, the implementation of NGS in routine labs is an important driver to reconsider the future role of NRCs. Molecular typing for public health surveillance is undergoing a stepwise transition to NGS [41]. Current and future NGS activities represented in this national survey were mainly in the context of food- and waterborne outbreak detections and investigations, reflecting the priority for these diseases across Europe and beyond [63, 64]. Several criteria should be considered in the process of integrating WGS in a routine laboratory setting [11] in order to know in which situations and for which pathogens it is worthwhile to use NGS. Identifying a set of key drivers that cover all aspects related to the implementation of NGS (utility and feasibility) can help to guide prioritization of pathogens and to efficiently allocate resources. Clinical and/or public health significance of the pathogen was scored as the most important driver during the implementation of pathogen genomics in routine public health activities, followed by availability of expertise to generate, analyze and interpret WGS data, and priority of the pathogen with respect to preventing the spread of antimicrobial resistance.

Qualitative responses revealed the opinion of several participants that the assessment of the added value of new technologies for individual patient care is paramount. If pathogen genomics is routinely used to guide patient management (diagnosis and/or treatment options), the pathogen sequence data gathered for diagnostic purposes can be accumulated for public health activities [65]. If there is no added value for routine diagnosis, the cost of WGS will have to be covered by limited public health budgets.

As a limitation, the relatively low response rate induced a potential volunteer bias as those public health experts being more interested and/or experienced in the field could be more likely to participate in the survey. Yet, 21% of the participants indicated that they were ‘not at all’ familiar with sequencing technologies and pathogen genomics. Further, we noticed a possible underrepresentation of the food, animal and environmental field in comparison to the human field, as well as a low number of bioinformaticians in the survey. In addition, public health professionals from the Belgian Institute for Health (Sciensano) might be overrepresented. The majority of microbiologists participating in the survey are based in a NRC, emphasizing surveillance activities and hence less weight to routine diagnostics. Given this potential imbalance, it is important to take into account the distribution of profiles within the study population while interpreting the results. However, it is difficult to ascertain the true underlying distribution of the different professional groups within the target population. Another limitation of the study is that the specific terminology used in the questions may not have been uniformly understood or consistently interpreted by stakeholders with different professional backgrounds [39].

## Conclusion

Public health professionals working in the field of infectious diseases in Belgium were in general enthusiastic about public health agencies implementing pathogen genomics for the surveillance and control of infectious diseases. However, introducing genomic methods into public health practice is inevitably linked to the decrease in cost, the introduction in routine activities of frontline clinical labs, the identification of field applications in public health, and the necessary development of new competencies. The results of the survey confirm the need to increase genomic literacy by offering dedicated training opportunities among public health professionals, especially for the data end-users including epidemiologists, clinicians, and infection control practitioners, enabling them to critically assess the utility and feasibility of implementing pathogen genomics in their work activities. As such, those at the forefront (i.e. end-users) may act as “honest brokers” responsible for evaluating the added value of genomic application. In the end, the main driver for the advancement of pathogen genomics in public health practice depends on the added value of this information for the different clinical and public health needs. Further, inter-disciplinary (between epidemiologists, microbiologists and bioinformaticians) and inter-sectoral (One Health context) collaboration should be improved in the future to pool expertise and to ensure an integrated and cohesive system for the management of infectious diseases. In terms of feasibility, respondents in this survey were mainly concerned, like their peers in similar European surveys, about data integration, data sharing, and the cost of sequencing technologies. Overall, this survey helps to better understand the perceived utility and feasibility of pathogen genomics according to public health professionals and can inform further guidance to facilitate its implementation in Belgium. Future challenges can be anticipated by performing a similar survey among public health experts based in a country that already progressed further in the process of implementing pathogen genomics within their public health surveillance system.

## Supplementary information

**Additional file 1.** “Questionnaire”. Description of data: “List of questions included in the online survey”.

**Additional file 2.** “Selection of target groups for the survey”. Description of data: “An overview of existing surveillance systems to identify all public health professionals who (would potentially) generate or use NGS data for the surveillance of infectious diseases based in different institutes and organizations in Belgium.”

**Additional file 3.** “Full responses to questionnaire”. Description of data: “Full responses to all questions included in the survey”.

**Additional file 4.** “Thematic analysis of open questions”. Description of data: “A full list of identified themes and the coded text”.

**Additional file 5.** “Word cloud based on open questions”. Description of data: “Word cloud constructed based on the free text responses visualizing the word frequency”.

## Data Availability

The raw data analyzed during the current study are available from the corresponding author on reasonable request.

## Abbreviations

AIC: Akaike Information Criterion
BAPCOC: Belgian Antibiotic Policy Coordination Committee
BICS: Belgian Infection Control Society
BSFM: Belgian Society for Food Microbiology
BVIKM: Belgian Society of Infection Specialists and Clinical Microbiologists
CI: Confidence interval
ECDC: European Centre for Disease Prevention and Control
EFSA: European Food Safety Authority
EU: European Union
FASFC: Federal Agency for the Safety of the Food Chain
MDR: Multidrug-resistant
NGS: Next generation sequencing
NRC: National Reference Centre
OR: Odds ratio
SNP: Single nucleotide polymorphism
WGS: Whole-genome sequencing
WHO: World Health Organization
