# Supplementary material for: Perceived utility and feasibility of pathogen genomics for public health practice: a survey among public health professionals working in the field of infectious diseases, Belgium, 2019
Source: BMC Public Health. 2020 Aug 31;20:1318. doi: 10.1186/s12889-020-09428-4 (PMC7456758; doi:10.1186/s12889-020-09428-4)
Supplement: Supplementary file 1 — Additional file 1. “Questionnaire”. Description of data: “List of questions included in the online survey”. [file 12889_2020_9428_MOESM1_ESM.pdf]

INTRODUCTION

Perceived utility and feasibility of pathogen genomics for public health practice

Context

The advent and continuous improvement of sequencing technologies, especially the shift to **next-generation sequencing (NGS)**, provides many opportunities for the management of **infectious diseases**.

Next generation sequencing (NGS) is a technique to rapidly determine a complete or specific part of a genome (i.e. the complete genetic composition of an organism) thanks to the parallel characterization of multiple DNA sequences. NGS makes **whole-genome sequencing (WGS)** accessible and is able to provide full genomic characteristics of the infectious isolates. Moreover, NGS is a universal test potentially replacing/complementing a series of traditional microbiological tests.

Pathogen genomics offers several advantages over conventional microbiological methods:

- Discriminate between pathogens with greater sensitivity and specificity
- Provide detailed characteristics of a pathogen, including identity, virulence determinants, drug resistance, and relatedness to other pathogens

During the last decade, NGS is no longer limited to research settings and is being rapidly translated into **public health practice**.

Definitions

NGS = Next-Generation Sequencing = rapid DNA-sequencing technology = high-throughput technology = massively parallel sequencing of small fragments of DNA

WGS = Whole Genome Sequencing = determining the complete DNA sequence of an organism's genome at a single time

Genomics = A genome is an organism's complete set of DNA, including all of its genes. Genomics involves the sequencing and analysis of genomes through uses of high throughput DNA sequencing (e.g. NGS) and bioinformatics to assemble and analyze the function and structure of entire genomes.

Focus of this survey

NGS offers new opportunities in research, individual patient care, and public health. Here, we **focus** on the implementation of NGS in a **routine public health setting** for the population-level management of infectious diseases.

Objectives of this survey

The key to success in **translating pathogen genomics into public health practice** is to demonstrate an added value by answering better to the needs and expectations from public health experts, both those in charge of national surveillance of infectious diseases and those involved in local infection control. This survey therefore aims to assess the **perceived utility and feasibility** of pathogen genomics for public health practice according to **public health professionals working in the field of infectious diseases**.

PARTICIPANT INFORMATION STATEMENT

1. What is this study about?

You are invited to take part in a voluntary research study to give your opinion concerning the utility and feasibility of pathogen genomics in a routine public health context.

By giving your consent to take part in this study you are telling us that you understand what you have read, agree to take part in the research study as outlined below, and agree to the use of your personal information as described.

2. Why have I been invited?

You have been invited to participate in this study because you are a public health professional working in the field of infectious diseases, which is broadly defined as being an microbiologist, molecular biologist, lab technician, epidemiologist, clinician, clinical biologists, infection control practitioner, hospital hygienist, risk manager, policy maker, food safety inspector, etc.

3. Who is running the study?

- Nina Van Goethem, PhD student involved in the .Be READY project, Sciensano.
- Herman Van Oyen, Head of Department Epidemiology and public health at Sciensano.
- Sophie Quoilin, Head of Epidemiology of infectious diseases at Sciensano.
- Nancy Roosens, Head of Transversal activities in applied genomics at Sciensano and coordinator of the .Be READY project.
- Sigrid De Keersmaecker, Head of Biotech Platform, Transversal activities in applied genomics at Sciensano
- Brecht Devleesschauwer, Epidemiology and public health at Sciensano
- Other partners involved in the .Be READY project.

The .Be READY project is internally funded by Sciensano.

4. What will I have to do?

You will be asked to complete an online questionnaire. The questionnaire will include a few demographic questions and questions to determine your attitudes and opinions concerning the use of pathogen genomics in a routine public health context.

5. How much of my time will the study take?

Depending on your level of familiarity with the topic, the questionnaire should take approximately between 15 to 45 minutes to complete.

6. Who can take part in the study?

This study is open to all public health professionals, including microbiologists, molecular biologists, lab technicians, epidemiologists, bioinformaticians, clinicians, infection control practitioners, risk managers, policy makers, food safety inspectors, etc. working in the field of infectious diseases. As you have been identified as belonging to this target group, you have been invited to participate to this survey.

You are welcome to invite other people, if they belong to the target group of this survey (i.e. public health professionals working in the field of infectious diseases), to participate in the study. However, only respondents with a token can participate. You can forward their email addresses to nina.vangoethem@sciensano.be and they will receive a personal invitation to the survey.

7. Do I have to be in the study? Can I withdraw from the study once I've started?

Participating in this study is completely voluntary. You can withdraw your responses any time before you have submitted the questionnaire.

8. Are there any risks or costs associated with being in the study?

Aside from giving your time, there will be no risks or costs associated with taking part in this study.

9. Are there any benefits associated with being in the study?

We aim to use the information from this study to further guide and facilitate the implementation of pathogen genomics in routine public health. If you are interested, you are welcome to leave your email address at the end of the survey and you will be informed about the results.

10. What will happen to information about me that is collected during the study?

The responses are anonymized. Tokens are used as it concerns a closed mode survey, however there is no connection between the token table and the data. The IP address will not be saved. Collected data will be stored securely and will be kept strictly confidential. Study findings may be published and/or presented in a variety of forms, but you will not be individually identifiable in these publications.

11. What if I would like further information about the study?

Nina Van Goethem will be available to further discuss this study with you and answer any questions you may have. If you would like to know more at any stage during the study, please send an email to nina.vangoethem@sciensano.be

You can access/print/save the summary (previous page) and the Participant Information Statement via this link.

○ I have read the Participant Information Statement and agree to take part in this study as outlined above

BACKGROUND

Institution/organization: who is your primary employer? *
Please select the answer that fits best.

Please choose **only one** of the following:
- Public sector (health authorities, scientific institution, etc.)
- Private sector
- Hospital (including University Hospitals)
- University
- Other

Which institute? *
Only answer this question if the following conditions are met:

Answer was 'Public sector (health authorities, scientific institution, etc.)' at question '2 [G1Q00002]' (Institution/organization: who is your primary employer? Please select the answer that fits best.)

Please choose **only one** of the following:
- Sciensano - Belgian institute for health
- Agentschap Zorg en Gezondheid (AZG)
- Agence pour une Vie de Qualité (AViQ)
- Commission communautaire commune de Bruxelles-Capitale (COCOM)
- Federal Overheidsdienst Volksgezondheid / Service Public Fédéral Santé Publique
- National Institute for Health and Disability Insurance (RIZIV/INAMI)
- Inter Mutualistic Agency (IMA-AIM)
- Belgian Healthcare Knowledge Centre (KCE)
- Superior Health Council
- Federal Agency for the Safety of the Food Chain (AFSCA-FAVV)
- Antimicrobial Consumption and Resistance in Animals (AMCRA)
- Other

What kind of hospital? *
Only answer this question if the following conditions are met:

Answer was 'Hospital (including University Hospitals)' at question '2 [G1Q00002]' (Institution/organization: who is your primary employer? Please select the answer that fits best.)

Please choose **only one** of the following:
- University hospital
- Private hospital
- Public hospital
- Other

What is your profession and/or your professional background? *
Please select the answer that fits best.

Please choose **only one** of the following:
- Microbiologist/laboratory scientist - directly involved in handling and/or testing specimes (pathogen expert)
- Molecular biologist - using advanced techniques to study organisms at the molecular level
- Clinical biologist - involved in clinical diagnostics (patient samples)
- Bioinformatician - developing computational approaches/algorithms to analyse genomic data from pathogens
- Epidemiologist/public health professional - work with data to understand patterns in disease occurrence (at the population level)
- Infection control practitioner - local prevention and control of infectious diseases in the community (frontline, including field epidemiologists)
- Hospital hygienist - prevention and control of healthcare associated infections (frontline, including field epidemiologists)

○Clinician: infectiology - directly involved in patient care and/or case management

○Clinician: other - directly involved in patient care and/or case management

○Public health policy maker - the making and implementing of decisions to improve the health of the public

○Food safety inspector - monitoring food products to ensure compliance with food safety standards

○Other

What kind of laboratory? *

Only answer this question if the following conditions are met:

Answer was 'Microbiologist/laboratory scientist - directly involved in handling and/or testing specimes (pathogen expert)' at question '5 [G1Q00001]' (What is your profession and/or your professional background? Please select the answer that fits best.)

Please choose **only one** of the following:

○National Reference Center (NRC) / National Reference Laboratory (NRL)

○Sentinel (periferal) laboratory

○Other

What is your age?

Please choose **only one** of the following:

○Under 25 years old

○25-34 years old

○35-44 years old

○45-54 years old

○55-64 years old

○65 years or older

How many years of professional experience do you have in the field of infectious diseases?*

Please choose **only one** of the following:

○No experience

○<1 year

○1-5 years

○6-10 years

○>10 years

What is your position within your institute/company?

Please choose **only one** of the following:

○Upper management (supervising multiple departments)

○Middle management (supervising multiple teams)

○Lower management (supervising a small team)

○Employee

○Other

Which discipline(s) is(are) your main field of action?*

Please choose **all** that apply:

☐Human

☐Animal

☐Food/feed

☐Environmental

Which pathogen(s) is(are) your main field of action?

Please choose **all** that apply:

☐Bacteria

☐Viruses

☐Parasites

☐Fungi/yeast

☐Other:

What group of infectious diseases do you work with? *

Please choose **all** that apply:

☐Respiratory infections (e.g. influenza)

☐Invasive bacterial diseases (e.g. N. meningitidis)

☐Vaccine preventable diseases (e.g. measles virus)

☐Consumption-related infectious diseases (food- and waterborne) (e.g. Salmonella)

☐Body-fluid related infectious diseases (e.g. HIV, Hepatitis, STI's)

☐Environmental-related diseases (zoonoses, vector-borne) (e.g. malaria)

☐Healthcare-associated infections (e.g. Clostridium difficile, MRSA)

☐Animal diseases

☐Other:

How familiar are you with Next Generation Sequencing (NGS) technologies and pathogen genomics? *

Please choose **only one** of the following:

○Very - I am involved in the generation and/or use of NGS data

○Somewhat - I have a general sense of the applications of NGS

○Not at all - I don't know anything about NGS and its applications

I am mainly involved/experienced with NGS in the following context:*

Only answer this question if the following conditions are met:

Answer was 'Very - I am involved in the generation and/or use of NGS data ' at question '13 [G1Q000010]' (How familiar are you with Next Generation Sequencing (NGS) technologies and pathogen genomics?)

Please choose **all** that apply:

☐NGS applied for sequencing parts of the genome

☐NGS applied for whole genome sequencing (WGS)

☐NGS applied for targeted metagenomics (e.g. 16S)

☐NGS applied for shotgun metagenomics

☐Other:

Given that I am not at all familiar with NGS and pathogen genomics ...*

Only answer this question if the following conditions are met:

Answer was 'Not at all - I don't know anything about NGS and its applications' at question '13 [G1Q000010]' (How familiar are you with Next Generation Sequencing (NGS) technologies and pathogen genomics?)

Please choose **only one** of the following:

○I want to know more, continue the survey and answer some general questions

○I prefer to quit the survey

ATTITUDE TOWARDS PATHOGEN GENOMICS FOR PUBLIC HEALTH PRACTICE

How enthusiastic are you about public health agencies using genomics to understand and control infectious diseases? *

Note: the focus of this survey is on the implementation of pathogen genomics in public health practice (infectious disease surveillance and control).

Only answer this question if the following conditions are met:

Answer was NOT 'I prefer to quit the survey' at question '15 [G1Q000015]' (Given that I am not at all familiar with NGS and pathogen genomics ...)

Please choose **only one** of the following:

- ○ Very enthusiastic - we should be using genomics now
- ○ Neutral - I do not have an opinion on genomics in public health
- ○ Skeptical - genomics may be useful for research purposes, but I do not see clear applications and/or an added value for public health
- ○ It's all a hype - genomics has not proven itself to be more useful than the conventional methods, we should not invest resources/time in genomics
- ○ I don't know - I don't know enough of the topic to be able to give an opinion

***Genomics** involves the sequencing and analysis of genomes (an organism's complete set of DNA/RNA) through uses of high throughput sequencing technologies [such as Next-Generation Sequencing (**NGS**)] and bioinformatics to assemble and analyze the function and structure of entire genomes [i.e. whole-genome sequencing (**WGS**)].*

How do you envision the impact* of pathogen genomics on public health practice in the near future (next 5 years) for the following activities? *

*If pathogen genomics will potentially have a major impact on a certain activity, this means that it has great added value (utility) and/or feasibility for this activity, i.e. it is likely that it will be implemented and replace/complement current methods in the next 5 years.

Only answer this question if the following conditions are met:

Answer was NOT 'I prefer to quit the survey' at question '15 [G1Q000015]' (Given that I am not at all familiar with NGS and pathogen genomics ...)

Please choose the appropriate response for each item:

	No impact	Minor impact	Major impact	I don't know
Patient management: Making a diagnosis (pathogen identification)	○	○	○	○
Patient management: Selecting an appropriate treatment	○	○	○	○
Outbreak investigations: Food/waterborne outbreak (source tracing, contact tracing)	○	○	○	○
Outbreak investigations: Nosocomial outbreak (source tracing, contact tracing)	○	○	○	○
Control-oriented surveillance: Identifying an outbreak (clusters of related isolates)	○	○	○	○
Strategy-oriented surveillance: Evaluating prevention and control programs (e.g. impact of vaccination programs)	○	○	○	○
Strategy-oriented surveillance: Monitoring the spread of antimicrobial resistance	○	○	○	○

Strategy-oriented surveillance: Monitoring the emergence and spread of zoonotic pathogens (integrated surveillance in both animal and human populations)				
Strategy-oriented surveillance: Source attribution (statistical modeling to attribute human cases to putative sources of infection)				

Outbreak investigations: to test epidemiological hypotheses of suspected outbreaks by retrospective (or close to real-time) comparisons of pathogens.

Control-oriented surveillance: to identify each occurrence of a particular disease, hazard, or other health-related event that requires a specific response, and to support the delivery of an effective intervention. For example, WGS-based continuous real-time surveillance for outbreak detection.

Strategy-oriented surveillance: to monitor long-term changes in epidemiology over larger geographic and population scales, requiring study designs that have a high degree of representativeness, to provide information to support prevention strategies. For example, cross-sectional genomic epidemiology surveys and sentinel surveillance.

Are there other public health activities in the field of infectious diseases not listed above that will benefit from the implementation of pathogen genomics, in your opinion?

Only answer this question if the following conditions are met:

Answer was NOT 'I prefer to quit the survey' at question '15 [G1Q000015]' (Given that I am not at all familiar with NGS and pathogen genomics ...)

Please write your answer here:

What are your main concerns about the implementation of pathogen genomics for public health practice (i.e. the main bottlenecks for routine implementation)? *

Only answer this question if the following conditions are met:

Answer was 'Somewhat - I have a general sense of the applications of NGS' *or* 'Very - I am involved in the generation and/or use of NGS data ' at question '13 [G1Q000010]' (How familiar are you with Next Generation Sequencing (NGS) technologies and pathogen genomics?)

Please choose the appropriate response for each item:

	Unconcerned	Somewhat unconcerned	Somewhat concerned	Very concerned	I don't know
Quality of the pathogen sequence data (validation and accreditation of both wet and dry lab protocols)					
Timeliness of the pathogen sequence data (turn-around time)					
Integration of pathogen sequence data with other types of data (e.g. clinical and epidemiological data)					
Linking pathogen sequence data from different sources (human/food/animal/environment)					
Translation of pathogen sequence data into public health action (usefulness)					
Interdisciplinary working/coordination between epidemiologists, microbiologists, bioinformaticians, etc.					
Cost of sequencing technologies					

Expertise and availability of personnel to be able to analyse pathogen sequence data (bioinformatics)					
Expertise and availability of personnel to be able to generate pathogen sequence data (wet lab)					
Timely and open sharing of pathogen sequence data and accompanying metadata					
Infrastructure (sequencers, high-performance computing, data storage, etc.)					
Availability of WGS typing schemes and reference databases (e.g. for antimicrobial resistance)					
Ethical and legal issues (e.g. patient privacy)					

What are your main concerns about the implementation of pathogen genomics for public health practice (i.e. the main bottlenecks for routine implementation)? *

Only answer this question if the following conditions are met:

Answer was 'Not at all - I don't know anything about NGS and its applications' at question '13 [G1Q000010]' (How familiar are you with Next Generation Sequencing (NGS) technologies and pathogen genomics?) *and* Answer was 'I want to know more, continue the survey and answer some general questions' at question '15 [G1Q000015]' (Given that I am not at all familiar with NGS and pathogen genomics ...)

Please choose the appropriate response for each item:

	Unconcerned	Somewhat unconcerned	Somewhat concerned	Very concerned	I don't know
Expertise of personnel to be able to generate, analyse, and interpret NGS data					
Availability of personnel to be able to generate, analyse, and interpret NGS data					
Cost of sequencing technologies					
Timeliness of the pathogen sequence data (turn-around time)					
Ethical and legal issues (e.g. patient privacy)					
Translation of pathogen sequence data into public health action (usefulness)					

Do you have any other concerns (bottlenecks to implementation in routine public health activities) not listed above?

Only answer this question if the following conditions are met:

Answer was NOT 'I prefer to quit the survey' at question '15 [G1Q000015]' (Given that I am not at all familiar with NGS and pathogen genomics ...)

Please write your answer here:

CONFIDENCE IN INTERPRETING NGS DATA

What is your training level in the field of genomics? *

Check <u>all</u> that apply: e.g. Masters in Biochemistry/Biotechnology/Molecular Biology/Bioinformatics AND Professional experience AND Continuing education.

Only answer this question if the following conditions are met:

Answer was NOT 'I prefer to quit the survey' at question '15 [G1Q000015]' (Given that I am not at all familiar with NGS and pathogen genomics ...)

Please choose **all** that apply:

☐None (I have never followed any training in genomics/genetics/molecular biology/bioinformatics)

☐Undergraduate (short courses on genomics/genetics/molecular biology/bioinformatics)

☐Masters in Biochemistry/Biotechnology/Molecular Biology/Bioinformatics or a related field

☐PhD in Biochemistry/Biotechnology/Molecular biology/Bioinformatics or a related field

☐Professional experience

☐Continuing education (attending workshops, trainings, self-directed learning, etc.)

☐Other:

What are the main reasons for not following any training/courses in genomics/genetics/molecular biology? *

Only answer this question if the following conditions are met:

Answer was 'None (I have never followed any training in genomics/genetics/molecular biology/bioinformatics)' at question '22 [G3Q00001]' (What is your training level in the field of genomics? Check all that apply: e.g. Masters in Biochemistry/Biotechnology/Molecular Biology/Bioinformatics AND Professional experience AND Continuing education.)

Please choose **all** that apply:

☐Lack of time

☐Lack of budget

☐Lack of available/suitable trainings/courses

☐Other:

Do you feel the need and/or would you be interested in following (additional) courses/training/workshops covering a topic related to pathogen genomics? *

Only answer this question if the following conditions are met:

Answer was NOT 'I prefer to quit the survey' at question '15 [G1Q000015]' (Given that I am not at all familiar with NGS and pathogen genomics ...)

Please choose **only one** of the following:

◯Yes

◯No

Please indicate on a scale from 1 to 5 how confident you are in working with the following data formats or output: *

1 = not confident at all
5 = highly confident

Only answer this question if the following conditions are met:

Answer was 'Somewhat - I have a general sense of the applications of NGS' *or* 'Very - I am involved in the generation and/or use of NGS data ' at question '13 [G1Q000010]' (How familiar are you with Next Generation Sequencing (NGS) technologies and pathogen genomics?)

Please choose the appropriate response for each item:

	1	2	3	4	5	I have no idea what this is
FASTQ: text-based format for storing raw unaligned sequences and their corresponding quality scores	○	○	○	○	○	○
BAM/SAM (Sequence Alignment/Map): aligned sequences	○	○	○	○	○	○
GFF/GTF and BED: formats for annotation with information and scores	○	○	○	○	○	○
VCF (Variant Call Format): text file for storing gene sequence variations	○	○	○	○	○	○
Data on resistance-conferring mutations present in the genome (e.g. ResFinder)	○	○	○	○	○	○
Phylogeny inferred from Single Nucleotide Polymorphisms (SNPs) (e.g. CSIPhylogeny)	○	○	○	○	○	○
wgMLST/cgMLST profiles	○	○	○	○	○	○
SNP address nomenclature	○	○	○	○	○	○

CURRENT AND FORESEEN NGS ACTIVITIES

Are you currently using/generating NGS data for any pathogen(s)? *

Only answer this question if the following conditions are met:

Answer was 'Very - I am involved in the generation and/or use of NGS data ' *or* 'Somewhat - I have a general sense of the applications of NGS' at question '13 [G1Q000010]' (How familiar are you with Next Generation Sequencing (NGS) technologies and pathogen genomics?)

Please choose **only one** of the following:

○ Yes

○ No

If yes, for which pathogen groups and purposes?

Only answer this question if the following conditions are met:

Answer was 'Yes' at question '26 [G9Q00001]' (Are you currently using/generating NGS data for any pathogen(s)?)

	Patient management (diagnosis and/or treatment options)	Outbreak investigations	Control-oriented surveillance	Strategy-oriented surveillance
Respiratory infections (e.g. influenza)	☐	☐	☐	☐
Invasive bacterial disease (e.g. N. meningitidis)	☐	☐	☐	☐
Vaccine preventable diseases (e.g. measles virus)	☐	☐	☐	☐
Food- and waterborne infectious diseases (e.g. Salmonella)	☐	☐	☐	☐
Body-fluid related infectious diseases (e.g. HIV, Hepatitis, STI's)	☐	☐	☐	☐
Environmental-related diseases (zoonoses, vector-borne) (e.g. malaria)	☐	☐	☐	☐
Healthcare-associated infections (e.g. Clostridium difficile, MRSA)	☐	☐	☐	☐
Animal diseases	☐	☐	☐	☐

Outbreak investigations: to test epidemiological hypotheses of suspected outbreaks by retrospective (or close to real-time) comparisons of pathogens.

Control-oriented surveillance: to identify each occurrence of a particular disease, hazard, or other health-related event that requires a specific response, and to support the delivery of an effective intervention. For example, WGS-based continuous real-time surveillance for outbreak detection.

Strategy-oriented surveillance: to monitor long-term changes in epidemiology over larger geographic and population scales, requiring study designs that have a high degree of representativeness, to provide information to support prevention strategies. For example, cross-sectional genomic epidemiology surveys and sentinel surveillance.

If possible, can you specify for which pathogens in particular you are generating/using NGS data?

Only answer this question if the following conditions are met:

Answer was 'Yes' at question '26 [G9Q00001]' (Are you currently using/generating NGS data for any pathogen(s)?)

Please write your answer here:

If yes, which strains are subjected to NGS analysis (sampling fraction of the total number of collected strains)? *

Please indicate for which pathogen(s) in the comments, e.g.:
- All strains: Listeria
- Subset of strains (minority): Salmonella spp.

Only answer this question if the following conditions are met:

Answer was 'Yes' at question '26 [G9Q00001]' (Are you currently using/generating NGS data for any pathogen(s)?)

Comment only when you choose an answer.
Please choose all that apply and provide a comment:

☐All strains

☐Subset of strains: majority

☐Subset of strains: minority

☐I don't know

Indicate for which pathogen(s) in the comments

If yes, is NGS used as a replacement for traditional methods or as a complementary tool? *

Please indicate for which pathogen(s) in the comments.

Only answer this question if the following conditions are met:

Answer was 'Yes' at question '26 [G9Q00001]' (Are you currently using/generating NGS data for any pathogen(s)?)

Comment only when you choose an answer.
Please choose all that apply and provide a comment:

☐Replacement

☐Complementary

☐I don't know

Indicate for which pathogen(s) in the comments

Are you planning to use/generate NGS data for any (additional) pathogen(s) within **the next 3 years**?*

Only answer this question if the following conditions are met:

Answer was 'Somewhat - I have a general sense of the applications of NGS' *or* 'Very - I am involved in the generation and/or use of NGS data ' at question '13 [G1Q000010]' (How familiar are you with Next Generation Sequencing (NGS) technologies and pathogen genomics?)

Please choose **only one** of the following:

○Yes

○No

If yes, for which pathogen groups and purposes?

Only answer this question if the following conditions are met:

Answer was 'Yes' at question '31 [G9Q00003]' (Are you planning to use/generate NGS data for any (additional) pathogen(s) within the next 3 years?)

	Patient management (diagnosis and/or treatment options)	Outbreak investigations	Control-oriented surveillance	Strategy-oriented surveillance
Respiratory infections (e.g. influenza)	☐	☐	☐	☐
Invasive bacterial disease (e.g. N. meningitidis)	☐	☐	☐	☐
Vaccine preventable diseases (e.g. measles virus)	☐	☐	☐	☐
Food- and waterborne infectious diseases (e.g. Salmonella)	☐	☐	☐	☐
Body-fluid related infectious diseases (e.g. HIV, Hepatitis, STI's)	☐	☐	☐	☐
Environmental-related diseases (zoonoses, vector-borne) (e.g. malaria)	☐	☐	☐	☐
Healthcare-associated infections (e.g. Clostridium difficile, MRSA)	☐	☐	☐	☐
Animal diseases	☐	☐	☐	☐

If possible, can you specificy for which pathogens in particular it is planned to generate/use NGS data within the next 3 years?

Only answer this question if the following conditions are met:

Answer was 'Yes' at question '31 [G9Q00003]' (Are you planning to use/generate NGS data for any (additional) pathogen(s) within the next 3 years?)

Please write your answer here:

If not, are there any particular reasons why the implementation of NGS would not be an option?

Only answer this question if the following conditions are met:

Answer was 'No' at question '31 [G9Q00003]' (Are you planning to use/generate NGS data for any (additional) pathogen(s) within the next 3 years?)

Please write your answer here:

KEY DRIVERS

Which criteria (key drivers) would mainly trigger/advocate the implementation of pathogen genomics in routine public health activities? *

Please assign a weight between 1 and 5 to each criteria, according to the relative importance of this criteria for the prioritisation of pathogens concerning the implementation of pathogen genomics.

1 = this criteria is **not an important driver** during the decision process of implementing NGS for a certain pathogen
5 = this criteria is a **highly important driver** during the decision process of implementing NGS for a certain pathogen
Below, you have the possibility to indicate whether the score (importance) given to a criteria depends on having a particular pathogen in mind.

Only answer this question if the following conditions are met:

Answer was 'Somewhat - I have a general sense of the applications of NGS' *or* 'Very - I am involved in the generation and/or use of NGS data ' at question '13 [G1Q000010]' (How familiar are you with Next Generation Sequencing (NGS) technologies and pathogen genomics?)

Please choose the appropriate response for each item:

	1	2	3	4	5	I don't know
Clinical and/or public health significance	○	○	○	○	○	○
Priority with respect to preventing the spread of antimicrobial resistance	○	○	○	○	○	○
Local/national/international policy surveillance priorities or obligations	○	○	○	○	○	○
Importance of prevention and control programs (e.g. vaccination)	○	○	○	○	○	○
Utility of WGS for diagnostics and/or treatment decisions (individual patient care)	○	○	○	○	○	○
Utility of increased resolution to infer relatedness that would not be obtained via conventional methods	○	○	○	○	○	○
Utility of additional information on the virulome/resistome that would be obtained via conventional methods	○	○	○	○	○	○
Availability of high-quality/complete/standardized epidemiological and/or clinical data to provide context to the WGS results	○	○	○	○	○	○
Possibility to link genomic data from different sources (food-animal-human-environment)	○	○	○	○	○	○
Cost-effectiveness (e.g. replacing multiple tests)	○	○	○	○	○	○
Time-saving compared to conventional testing methods	○	○	○	○	○	○
Impact on outcomes for patients and populations (translation into actionable results)	○	○	○	○	○	○
Availability of WGS typing schemes and reference databases (e.g. for antimicrobial resistance)	○	○	○	○	○	○
Availability of validated (quality-controlled) WGS workflows (both wet and dry laboratory)	○	○	○	○	○	○

Availability of expertise to generate, analyze and interpret WGS data	○	○	○	○	○	○
Availability of the appropriate infrastructure (sequence technology, high-performance computing, data storage, etc.)	○	○	○	○	○	○

Would you rate one or more of the criteria (drivers) indicated above differently **according to the pathogen** you have in mind? Would the importance of the criteria depend on the pathogen? If applicable, please specify which criteria, and how its importance would change depending on the pathogen.

Only answer this question if the following conditions are met:

Answer was 'Somewhat - I have a general sense of the applications of NGS' *or* 'Very - I am involved in the generation and/or use of NGS data ' at question '13 [G1Q000010]' (How familiar are you with Next Generation Sequencing (NGS) technologies and pathogen genomics?)

Please write your answer here:

Are there any **additional criteria** (key drivers) worth considering for the decision of implementing next-generation sequencing (NGS) for public health activities related to a particular pathogen?

Only answer this question if the following conditions are met:

Answer was 'Somewhat - I have a general sense of the applications of NGS' *or* 'Very - I am involved in the generation and/or use of NGS data ' at question '13 [G1Q000010]' (How familiar are you with Next Generation Sequencing (NGS) technologies and pathogen genomics?)

Please write your answer here:

EXPECTED OUTPUT

The output from WGS needs to be in a format that is useful for its end-users. In order to facilitate the translation of complex genomic data into actionable results, which kind of **data formats** should be delivered following NGS analyses? *

In case this question is not applicable to you, i.e. you are not a (potential) end-user of NGS data, you can indicate 'I don't know/not applicable'

Please specify for which pathogens/applications in the comments.

Only answer this question if the following conditions are met:

Answer was 'Somewhat - I have a general sense of the applications of NGS' *or* 'Very - I am involved in the generation and/or use of NGS data ' at question '13 [G1Q000010]' (How familiar are you with Next Generation Sequencing (NGS) technologies and pathogen genomics?)

Please choose all that apply and provide a comment:

☐Whole-genome phylogenetic tree (identification of clusters)

☐Whole-genome phylogenetic tree combined with epidemiological links/exposures

☐Table on clustered cases

☐Line listings of resistance/virulence-conferring mutations (virulome/resistome)

☐Line listings of isolate pathovar name - antibiogramme profile

☐I don't know/not applicable

☐Other:

Please clarify for which pathogens/applications in the comments.

What would be the desired **turn-around time**? *

In case this question is not applicable to you, i.e. you are not a (potential) end-user of NGS data, you can indicate 'I don't know/not applicable'

Please specify for which pathogens/applications in the comments.

Only answer this question if the following conditions are met:

Answer was 'Somewhat - I have a general sense of the applications of NGS' *or* 'Very - I am involved in the generation and/or use of NGS data ' at question '13 [G1Q000010]' (How familiar are you with Next Generation Sequencing (NGS) technologies and pathogen genomics?)

Comment only when you choose an answer.
Please choose all that apply and provide a comment:

☐< one week

☐< one month

☐< three months

☐< one year

☐I don't know/not applicable

☐Other:

Please specify for which pathogens and/or specific situations in the comments.

DIAGNOSTIC HIERARCHY

Which WGS provision model seems most appropriate in the Belgian context, in your opinion? *

Only answer this question if the following conditions are met:

Answer was 'Very - I am involved in the generation and/or use of NGS data ' *or* 'Somewhat - I have a general sense of the applications of NGS' at question '13 [G1Q000010]' (How familiar are you with Next Generation Sequencing (NGS) technologies and pathogen genomics?)

Please choose **only one** of the following:

- Centralization of sequencing and bioinformatics at one central sequencing center
- Centralization of sequencing and bioinformatics at National Reference Centers (which are organized per pathogen or group of pathogens)
- Decentralized point-of-care sequencing (at frontline laboratories), but centralization of bioinformatics (mixed model)
- Decentralization of sequencing and bioinformatics, but mandatory submission of isolates to a national repository
- Decentralization of sequencing and bioinformatics, but mandatory submission of raw sequence data to a national repository
- Decentralization of sequencing and bioinformatics, but mandatory submission of bioinformatics output to a national repository
- I don't know
- Other

DATA SHARING

Rapid data sharing is critical during an unfolding health emergency. Therefore, public access to sequence data to inform public health activities is strongly recommended by WHO (https://www.who.int/blueprint/what/norms-standards/gsdsharing/en/).

What are according to you the main obstacles for sharing pathogen sequence data and associated metadata? *

Only answer this question if the following conditions are met:

Answer was 'Very - I am involved in the generation and/or use of NGS data ' *or* 'Somewhat - I have a general sense of the applications of NGS' at question '13 [G1Q000010]' (How familiar are you with Next Generation Sequencing (NGS) technologies and pathogen genomics?)

Please choose the appropriate response for each item:

	Major obstacle	Minor obstacle	No obstacle	I don't know
Practical barriers: lack of data standardization, poor data quality, missing meta-data, etc.	◯	◯	◯	◯
Political sensitivities: potential misuse of data, national security and safety, economic risks, etc.	◯	◯	◯	◯
Ethical issues and concerns: accompanying patient data might give rise to privacy issues (personal data protection)	◯	◯	◯	◯
Priority to publication: concerns over misuse and inability to publish	◯	◯	◯	◯

Are there any additional obstacles for sharing pathogen sequence data and associated metadata not mentioned above, and/or would you like to make some comments?

Only answer this question if the following conditions are met:

Answer was 'Very - I am involved in the generation and/or use of NGS data ' *or* 'Somewhat - I have a general sense of the applications of NGS' at question '13 [G1Q000010]' (How familiar are you with Next Generation Sequencing (NGS) technologies and pathogen genomics?)

Please write your answer here:

THANK YOU

Thank you for taking the time to complete this survey! Your responses will contribute to reach the objectives of the **.Be READY** project.

If you have any comments on the survey or the project, please leave a comment below. You are also welcome to leave your email address to receive feedback on the survey results.

Please write your answer here:
