# Supplementary material for: Perceived utility and feasibility of pathogen genomics for public health practice: a survey among public health professionals working in the field of infectious diseases, Belgium, 2019
Source: BMC Public Health. 2020 Aug 31;20:1318. doi: 10.1186/s12889-020-09428-4 (PMC7456758; doi:10.1186/s12889-020-09428-4)
Supplement: Supplementary file 2 — Additional file 2. “Selection of target groups for the survey”. Description of data: “An overview of existing surveillance systems to identify all public health professionals who (would potentially) generate or use NGS data for the surveillance of infectious diseases based in different institutes and organizations in Belgium.” [file 12889_2020_9428_MOESM2_ESM.pdf]

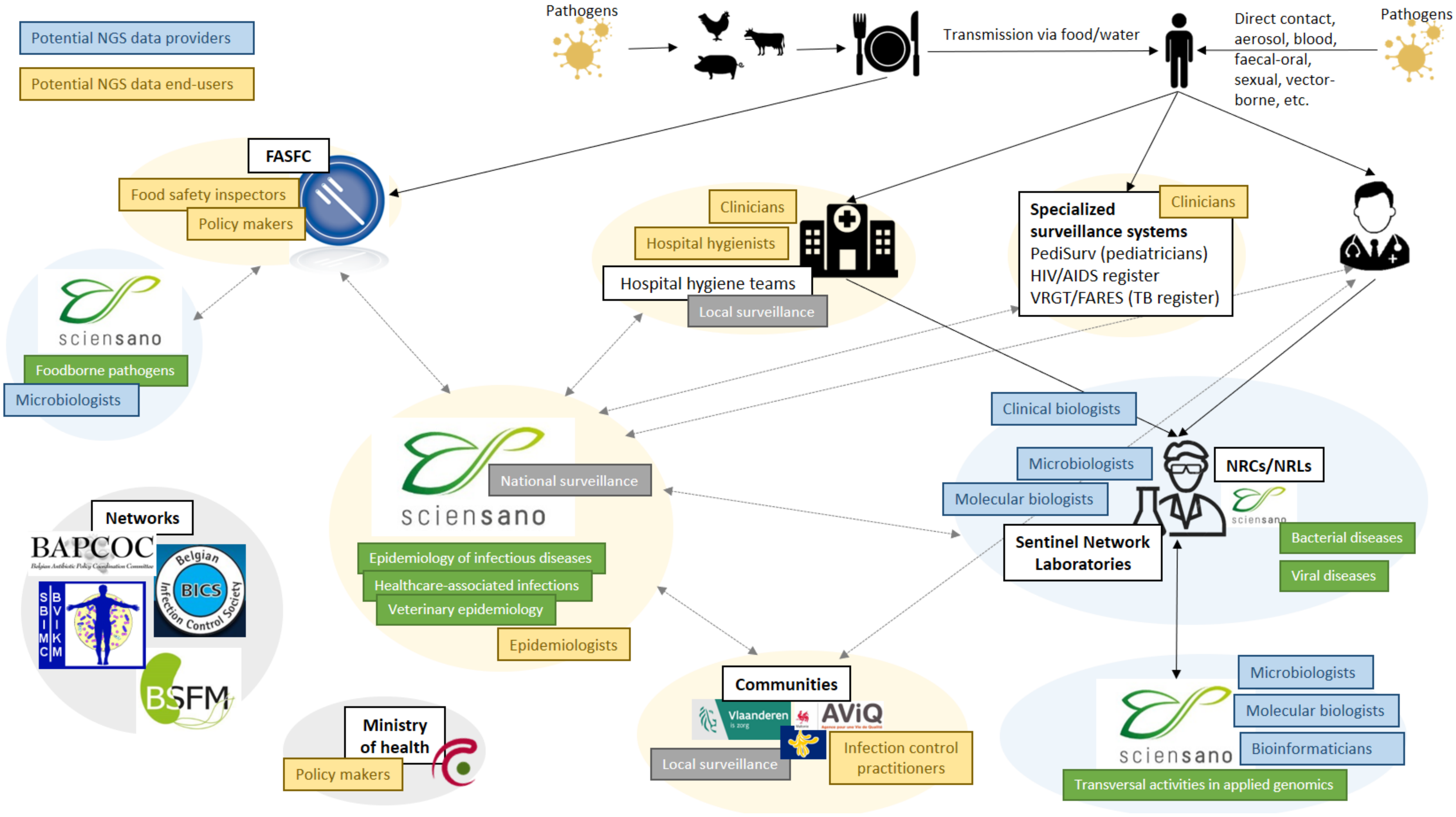

Potential NGS data providers
Potential NGS data end-users
Pathogens
Transmission via food/water
Direct contact, aerosol, blood, faecal-oral, sexual, vector-borne, etc.
Pathogens
FASFC
Food safety inspectors
Policy makers
sciensano
Foodborne pathogens
Microbiologists
Clinicians
Hospital hygienists
Hospital hygiene teams
Local surveillance
Specialized surveillance systems
PediSurv (pediatricians)
HIV/AIDS register
VRGT/FARES (TB register)
Clinicians
sciensano
National surveillance
Epidemiology of infectious diseases
Healthcare-associated infections
Veterinary epidemiology
Epidemiologists
Clinical biologists
Microbiologists
Molecular biologists
NRCs/NRLs
sciensano
Sentinel Network Laboratories
Bacterial diseases
Viral diseases
Networks
BAPCOC
Belgian Antibiotic Policy Coordination Committee
Belgian Infection Control Society
BICS
SBIMC
BVIKM
BSFM
Ministry of health
Policy makers
Communities
Vlaanderen is zorg
AViQ
Local surveillance
Infection control practitioners
Microbiologists
Molecular biologists
Bioinformaticians
sciensano
Transversal activities in applied genomics

Selection of target groups for the survey, Belgium, 2019.

A proportion of persons infected by a pathogen (through one of the different transmission routes, e.g. orally via food/water, aerosol, direct contact, …) will subsequently seek health care (e.g. hospital, general practitioner, …). A proportion of those will be registered through one of the different surveillance systems. Infectious disease surveillance is a multi-component system that monitors and analyzes data including demographic, geographic, and disease/condition-specific information. When a sample is taken, the specific characteristics of the infectious agent can be investigated as well, which complements the clinical and demographic patient information. Next-generation sequencing (NGS) allows pathogen identification and characterization with superior resolution compared to the conventional methods.

An overview was made of existing surveillance systems to identify all public health professionals who (would potentially) generate or use NGS data for the surveillance of infectious diseases based in different institutes and organizations in Belgium. It includes clinicians, who are at the frontline through identification of infected patients; microbiologists, who are involved in testing specimens after collecting samples from the patient/food/animal; molecular biologists, who study micro-organisms at the molecular level; bioinformaticians, who develop computational approaches/algorithms to analyze genomic data; epidemiologists, who use the data to understand patterns in disease occurrence at the population level; infection control practitioners, who are responsible for local prevention and control of infectious diseases in the community; hospital hygienists, who are involved in the prevention and control of healthcare-associated infections; food safety inspectors, who monitor food products; etc. This overview also indicates the position in the information cycle of the different professionals as data providers (blue) defined here as experts in wet and dry lab procedures and (potentially) generating NGS data (including microbiologists, molecular biologists, clinical biologists, and bioinformaticians), and data end-users (yellow) defined here as using NGS data to improve their activities and implementing infection control measures (including epidemiologists, local infection control practitioners, hospital hygienists, clinicians, and food safety inspectors).

In order to reach this target group, we contacted clinicians, clinical biologists and hospital hygienists participating in hospital surveillance networks; clinicians participating to specific infectious disease surveillance networks (e.g. PediSurv and HIV/AIDS register); scientists (including epidemiologists, microbiologists, molecular biologists, and bioinformaticians) from the Belgian institute for Health (Sciensano) working in infectious disease departments (including ‘Epidemiology of infectious diseases’, ‘Healthcare-associated infections’, ‘Veterinary epidemiology’, ‘Bacterial diseases’, ‘Viral diseases’, ‘Transversal activities in applied genomics’, and ‘Foodborne pathogens’); food safety inspectors from the Federal Agency for the Safety of the Food Chain (FASFC); infection control practitioners of the regional infectious disease control teams from the three regions in Belgium (communities); microbiologists from National Reference Centers (NRCs) and from sentinel laboratories; and public health experts within the ministry of health. In addition, we invited members from the Belgian Society of Infection Specialists and Clinical Microbiologists (BVIKM), the Belgian Antibiotic Policy Coordination Committee (BAPCOC), the Belgian Infection Control Society (BICS), and the Belgian Society for Food Microbiology (BSFM) to participate in the survey.
