# Supplementary material for: Perceived utility and feasibility of pathogen genomics for public health practice: a survey among public health professionals working in the field of infectious diseases, Belgium, 2019
Source: BMC Public Health. 2020 Aug 31;20:1318. doi: 10.1186/s12889-020-09428-4 (PMC7456758; doi:10.1186/s12889-020-09428-4)
Supplement: Supplementary file 3 — Additional file 3. “Full responses to questionnaire”. Description of data: “Full responses to all questions included in the survey”. [file 12889_2020_9428_MOESM3_ESM.pdf]

Full responses to questionnaire

1.1.1. Background questions

Who is your primary employer?

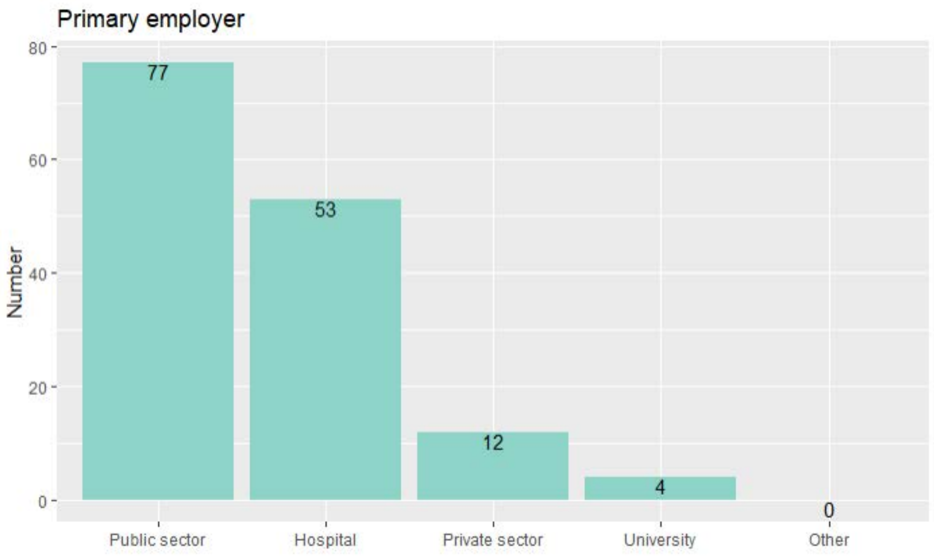

Which institute (only if indicated 'public sector')?

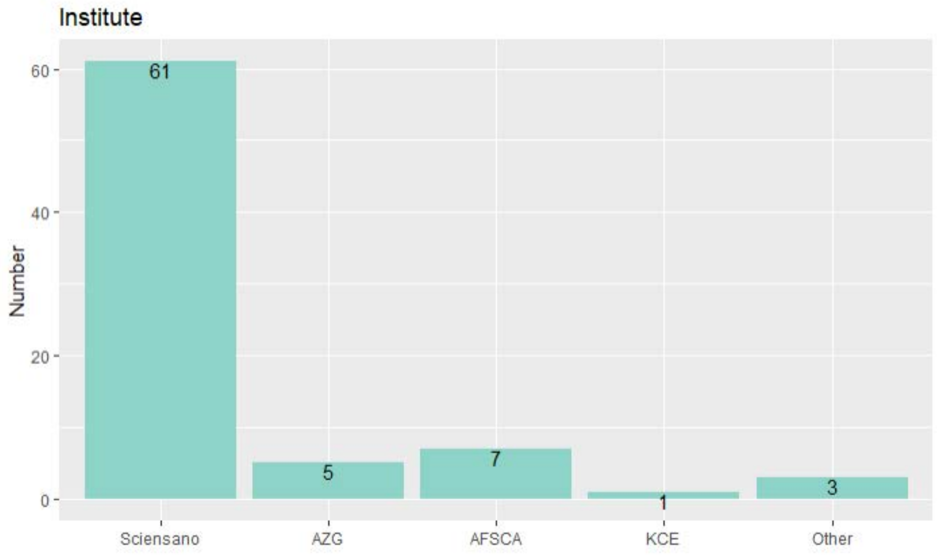

What kind of hospital (only if indicated 'hospital')?

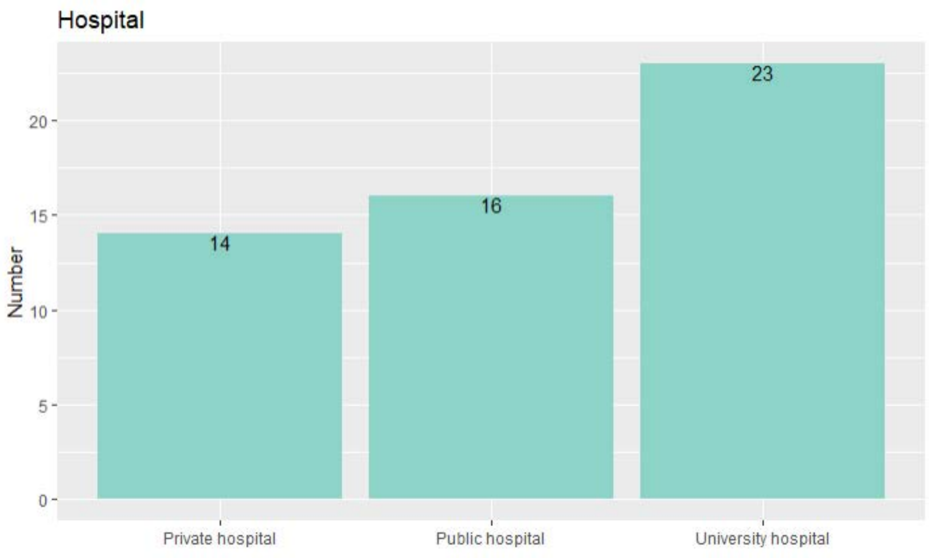

What is your profession and/or your professional background?

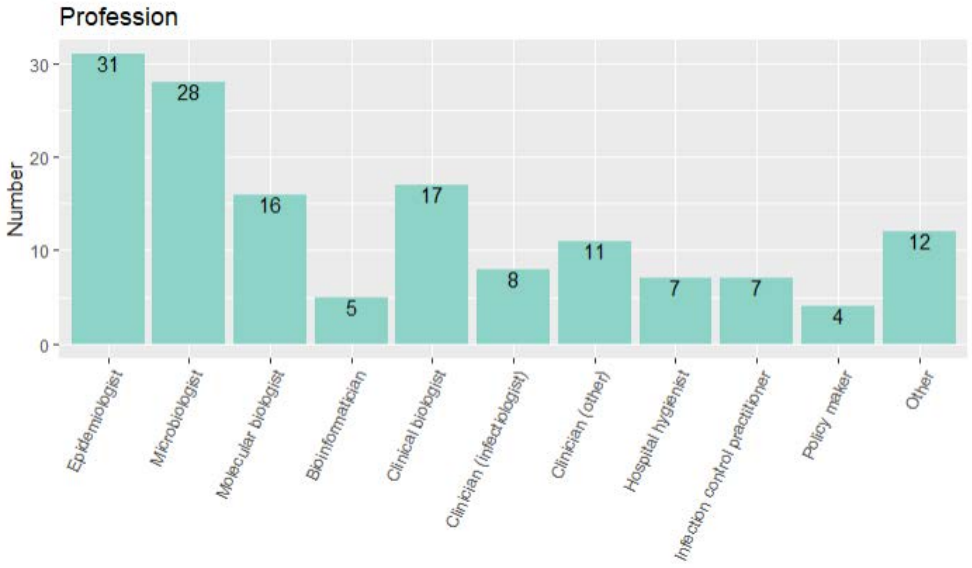

What is your age?

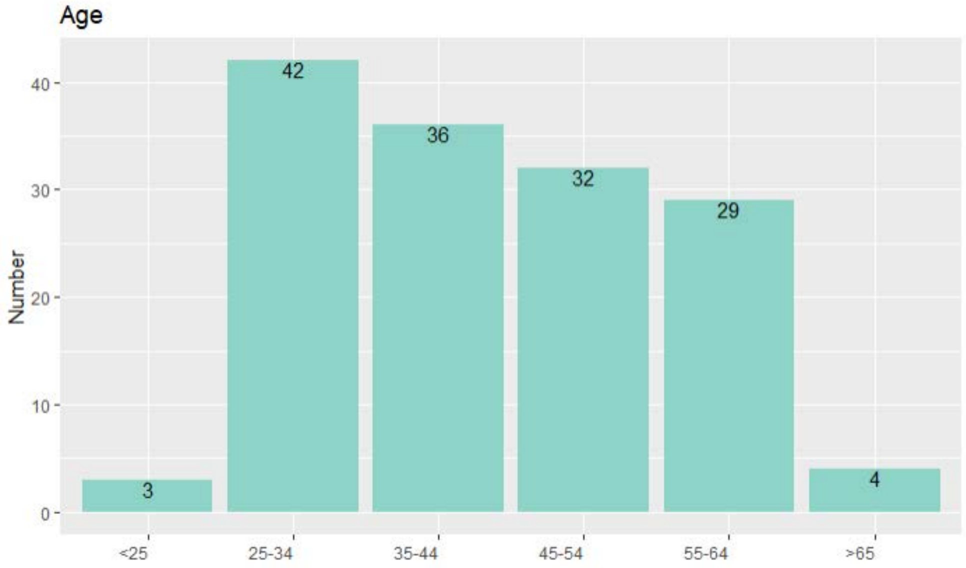

How many years of professional experience do you have in the field of infectious diseases?

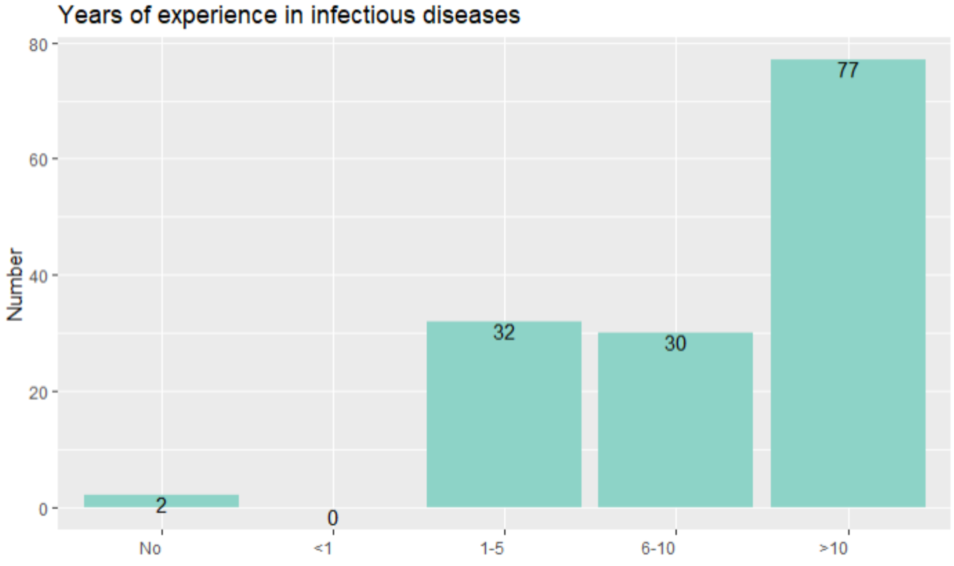

What is your position within your institute/company?

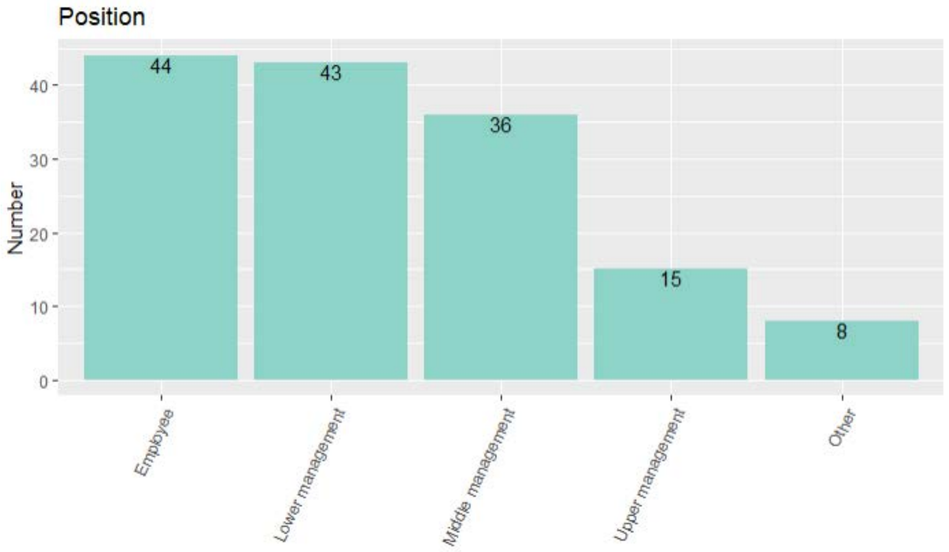

Which discipline(s) is (are) your main field of action (multiple choice is allowed)?

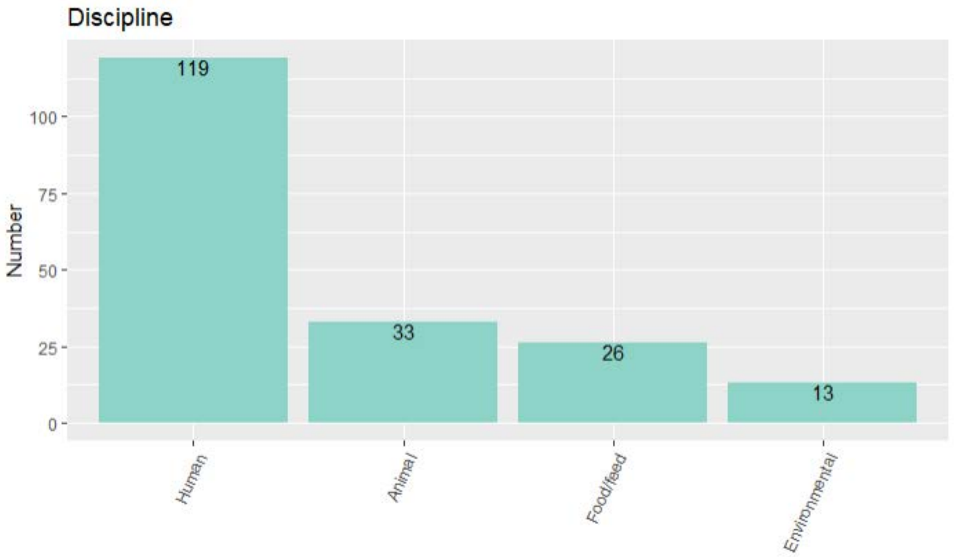

Which pathogen(s) is (are) your main field of action (multiple choice is allowed)?

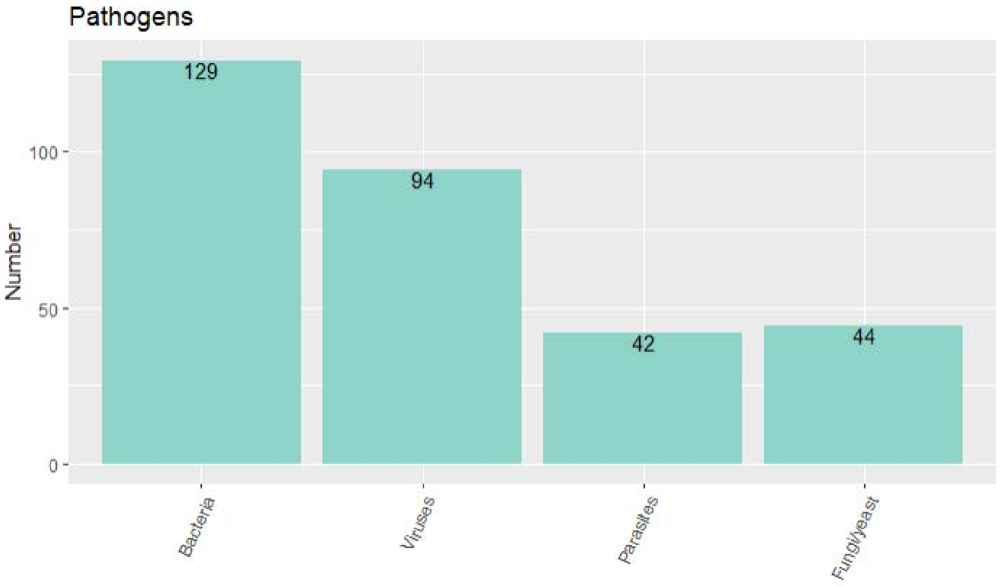

What group(s) of infectious diseases do you work with (multiple choice is allowed)?

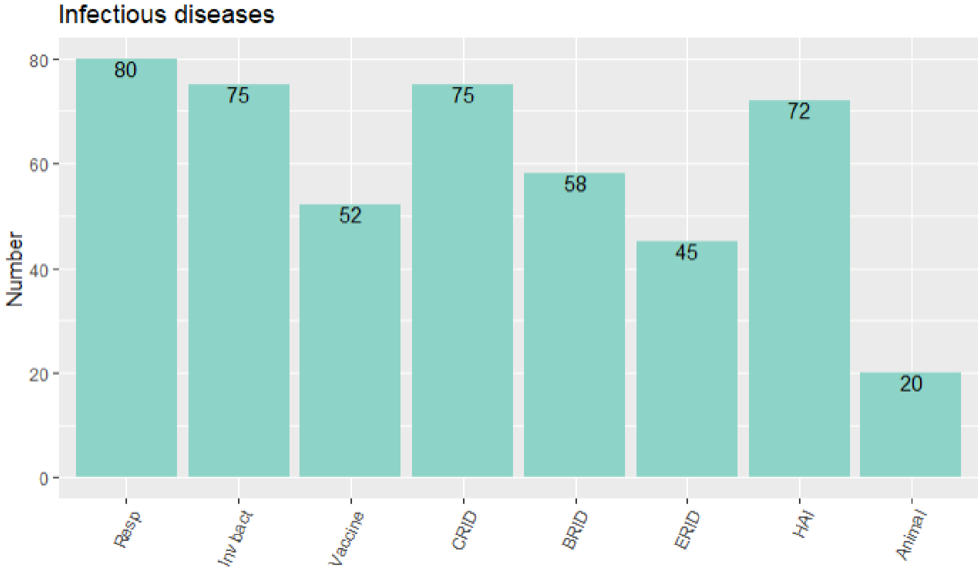

How familiar are you with Next-Generation Sequencing (NGS) technologies and pathogen genomics?

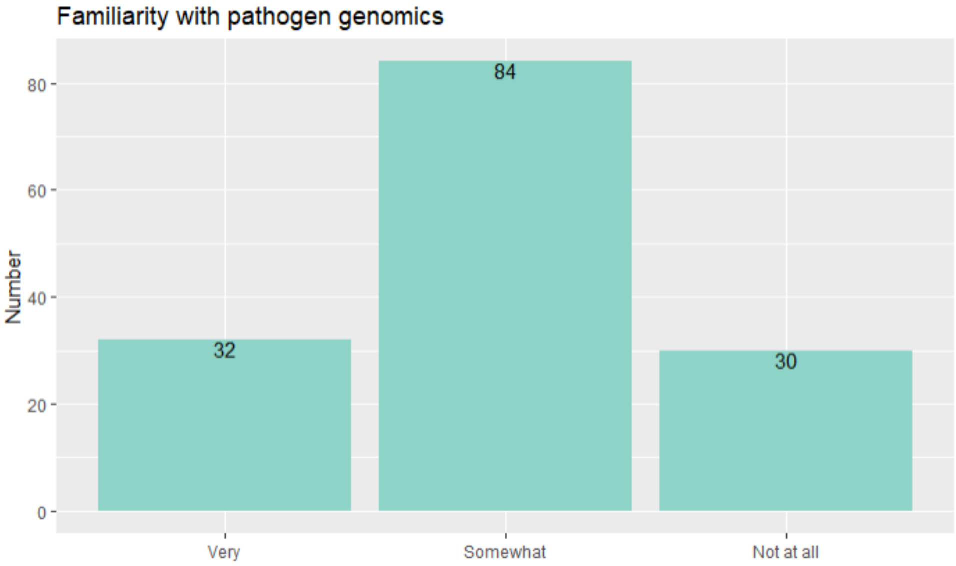

I am mainly involved/experienced with NGS in the following context (if answered 'very' familiar in previous question):

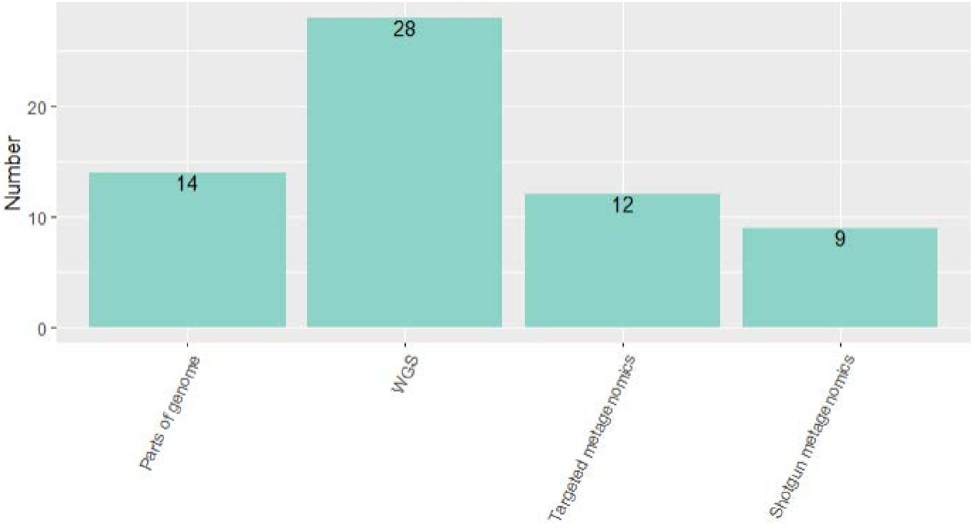

Given that I am not at all familiar with NGS and pathogen genomics (if answered 'not at all' in previous question):

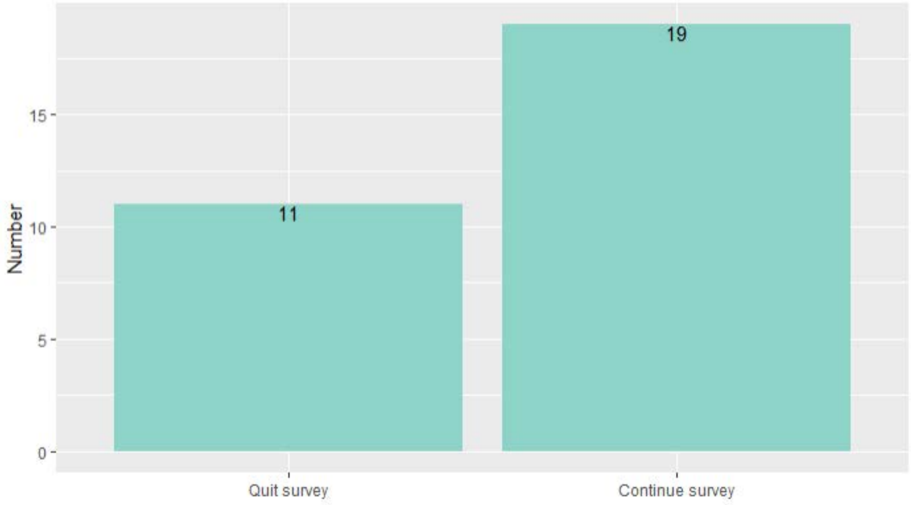

1.1.2. Attitude towards pathogen genomics for public health practice

How enthusiastic are you about public health agencies using genomics to understand and control infectious diseases?

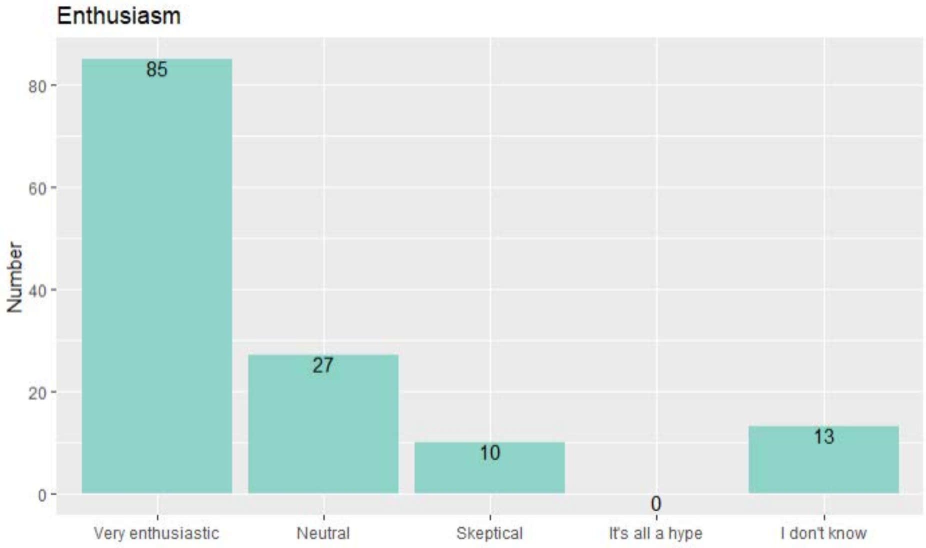

How do you envision the impact of pathogen genomics on public health practice in the near future (next 5 years) for the following activities (question for all participants, n=135)?

	Major impact	Minor impact	No impact	Don't know
Patient management: Making a diagnosis (pathogen identification)	44%	40%	8%	7%
Patient management: Selecting an appropriate treatment	44%	37%	10%	9%
Outbreak investigations: Food/waterborne outbreak (source tracing, contact tracing)	76%	15%	1%	7%
Outbreak investigations: Nosocomial outbreak (source tracing, contact tracing)	76%	12%	1%	10%
Control-oriented surveillance: Identifying an outbreak (clusters of related isolates)	78%	16%	2%	4%
Strategy-oriented surveillance: Evaluating prevention and control programs (e.g. impact of vaccination programs)	36%	36%	8%	20%

Strategy-oriented surveillance: Monitoring the spread of antimicrobial resistance	71%	19%	3%	7%
Strategy-oriented surveillance: Monitoring the emergence and spread of zoonotic pathogens (integrated surveillance in both animal and human populations)	54%	27%	5%	14%
Strategy-oriented surveillance: Source attribution (statistical modelling to attribute human cases to putative sources of infection)	46%	27%	5%	22%

Are there other public health activities in the field of infectious diseases not listed above that will benefit from the implementation of pathogen genomics, in your opinion?

- ‘A 5 year delay is probably too short, it will be not yet done in routine, it will be more for research.’
- ‘Discovery of causal relation between a pathogen and a clinical disease (e.g. cancer).’
- ‘Early diagnostics of diseases due to slowly growing pathogens (Tuberculosis) and early identification/diagnosis of emerging diseases.’
- ‘General characterization of pathogens.’
- ‘Identification and characterization of new strains.’
- ‘Identification of new clones.’
- ‘Pathogenicity (identification of virulence factors)’
- ‘Influence on appropriate antibiotic use or no use and diminishing the overall rate of resistance, control on antibiotic use in livestock breeding’
- ‘Drinking water quality’
- ‘Air quality, home environmental quality (moulds, yeasts)’
- ‘Insight in dysbiosis’
- ‘International tracking’
- ‘Vaccine development’
- ‘Phage therapy’
- ‘Metagenomics in clinical practice will not be implemented in little to moderate hospitals within the first 5y I'm afraid. Sciensano can offer solutions.’
- ‘Monitoring of antiviral resistance, monitoring of homology with vaccine strains (Influenza).’
- ‘Monitoring the emergence and spread of zoonotic pathogens has been impacted negatively, by the introduction of WGS at the human site only: I don't see this changing in the next 5 years unfortunately.’
- ‘To identify the possible origin of a bacteria or virus: whether it was imported or circulated within Belgium’
- ‘Monitoring trends in resistance of HIV (already widely used for years at the moment of diagnosis of HIV infection) and phylogenetic analysis to better understand the spread of the HIV infection.’
- ‘Monitoring of antiviral resistance, monitoring of homology with vaccine strains (influenza).’
- ‘Phylogenetic studies in an epidemiological context.’
- ‘Pathogens discovery (molecular identification and characterization of new putative pathogens).’

What are your main concerns about the implementation of pathogen genomics for public health practice (i.e. the main bottlenecks for routine implementation) (question for participants being ‘very’ or ‘somewhat’ familiar with pathogen genomics, n=116)?

	**Very concerned**	**Somewhat concerned**	**Somewhat unconcerned**	**Unconcerned**	**Don't know**
Quality of the pathogen sequence data (validation and accreditation of both wet and dry lab protocols)	23%	40%	15%	7%	16%
Timeliness of the pathogen sequence data (turn-around time)	25%	42%	20%	3%	9%

Integration of pathogen sequence data with other types of data (e.g. clinical and epidemiological data)	32%	38%	16%	5%	9%
Linking pathogen sequence data from different sources (human/food/animal/environment)	24%	35%	20%	10%	10%
Translation of pathogen sequence data into public health action (usefulness)	32%	36%	20%	5%	7%
Interdisciplinary working/coordination between epidemiologists, microbiologists, bioinformaticians, etc.	33%	42%	19%	4%	2%
Cost of sequencing technologies	54%	32%	8%	3%	3%
Expertise and availability of personnel to be able to generate pathogen sequence data (wet lab)	26%	34%	21%	9%	9%
Expertise and availability of personnel to be able to analyse pathogen sequence data (bioinformatics)	41%	34%	14%	3%	8%
Timely and open sharing of pathogen sequence data and accompanying metadata	49%	32%	9%	3%	7%
Infrastructure (sequencers, high-performance computing, data storage, etc.)	34%	34%	17%	5%	10%
Availability of WGS typing schemes and reference databases (e.g. for antimicrobial resistance)	28%	46%	15%	2%	10%
Ethical and legal issues (e.g. patient privacy)	23%	31%	24%	14%	8%

What are your main concerns about the implementation of pathogen genomics for public health practice (i.e. the main bottlenecks for routine implementation) (question for participants being 'not at all' familiar with pathogen genomics (n=19)?

	**Very concerned**	**Somewhat concerned**	**Somewhat unconcerned**	**Unconcerned**	**Don't know**
Expertise of personnel to be able to generate, analyse, and interpret NGS data	21%	32%	16%	16%	16%
Availability of personnel to be able to generate, analyse, and interpret NGS data	16%	37%	16%	11%	21%
Cost of sequencing technologies	53%	21%	11%	5%	5%
Timeliness of the pathogen sequence data (turn-around time)	42%	26%	5%	5%	21%
Ethical and legal issues (e.g. patient privacy)	16%	32%	21%	21%	11%
Translation of pathogen sequence data into public health action (usefulness)	21%	37%	16%	11%	16%

Do you have any other concerns (bottlenecks to implementation in routine public health activities) not listed above?:

- ‘Appropriate training of personnel for execution and interpretation.’
- ‘Data collection is already limited so newer technologies will not automatically improve this process but be redundant if the basics are not met.’
- ‘Does identification prove that the pathogen poses a risk? Risk is linked to quantity and establishing thresholds will take time. The number of bacteria, viruses etc. is infinite and we don't know yet the role (e.g. microbiome) of all of them, individual and together.’
- ‘Extremely important technology in which we should invest so that sufficient capacity is built. Standardization and facilities for data sharing need to be improved.’
- ‘Harmonization of epidemiological data. Most of the epidemiological data is very 'messy' or inconsistent, which makes systematic integration and surveillance unfeasible.’
- ‘How to interpret the result at clinical level.’
- ‘Implementation of the NGS results in the legislation and acceptance of the use of NGS for routine in enforcement laboratories by the competent authorities. For this they need to have a basic understanding (education) it order to understand and see cost/benefit of the whole picture (often the view is fragmented; analysis by analysis).’
- ‘Multidisciplinary knowledge of personal working on this topic.’
- ‘Reliability of generated data; high inter-laboratory variation.’
- ‘Storage of data - open access - international compatibilities - healthcare workers integrity concerns’
- ‘The only real and major concern is the fear that some actors in the field will try to abuse their power and influence to monopolize this new technology to only university hospitals or only to public health authorities like Sciensano. To really be valuable to patient management and public health it is absolutely required to offer free access to all Laboratories to this new technology.’
- ‘The exact topic of this survey, i.e. the perceived utility and feasibility of pathogen genomics by public health practitioners, which is the biggest bottleneck of all. All the other concerns listed above can be tackled given the drive within the field to solve them in the first place.’
- ‘In the HIV field, the phylogenetic analyses of virus permit to have an hindsight in paths of transmission. It is a very tricky topic in ethical and potentially legal aspects.’
- ‘Interpretation across sectors and communication within Belgium and across borders.’
- ‘Rapidity to obtain the sequences (difficulty to have it in real time), possibility to mix virus and bacteria to increase the rapidity to obtain results.’
- ‘Training’
- ‘The "kitome" problem, contaminated reagents kits, which becomes more and more clear while performing WGS/NGS in microbiology.’
- ‘The unit price for each analysis, although decreasing over time will be a limit and not all the labs could train and keep specialists in data analysis at work. The time limit for this vision (5years) might be too short... but with the automatization of the analysis process... it will become affordable.’
- ‘Who will sponsor all this?’

1.1.3. Confidence in interpreting NGS data

What is your training level in the field of genomics (multiple choice is allowed)?

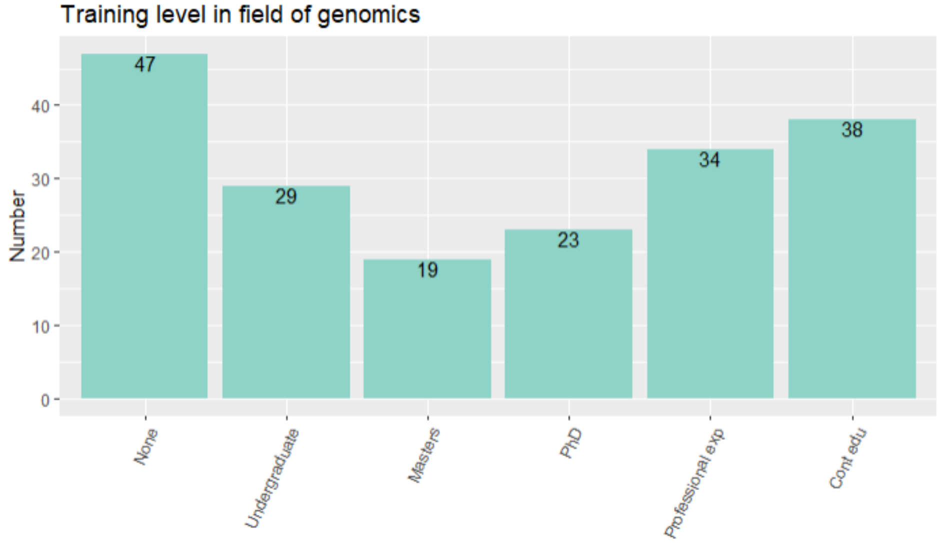

What are the main reasons for not following any training/courses in genomics/genetics/molecular biology?

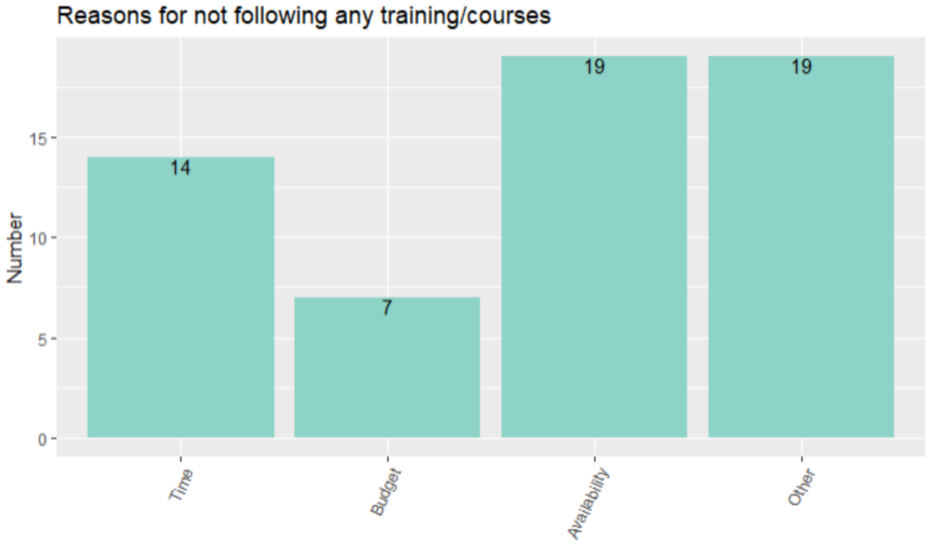

Other reasons:

- 'I'm a nurse infection prevention and this subject was never an issue in trainings or symposium.'
- 'I don't work in a lab.'
- 'Management position.'
- 'Lack of training adapted to public health needs.'
- 'Not applicable for a clinician.'
- 'Not my priority, others are already more qualified.'
- 'Not my field.'
- 'Not my role.'
- 'No need.'
- 'Not relevant for my practice.'
- 'No interest for my work/tasks.'
- 'Not my direct filed of practice (I'm a clinician).'
- 'Not priority for my current job.'
- 'Only following what matters to the own work field (clinician).'
- 'Not a priority in my profession.'
- 'Not directly my job, but interested.'
- 'Not really necessary actually in my daily work.'

- ‘Other priorities.’

Do you feel the need and/or would you be interested in following (additional) courses/training/workshops covering a topic related to pathogen genomics?

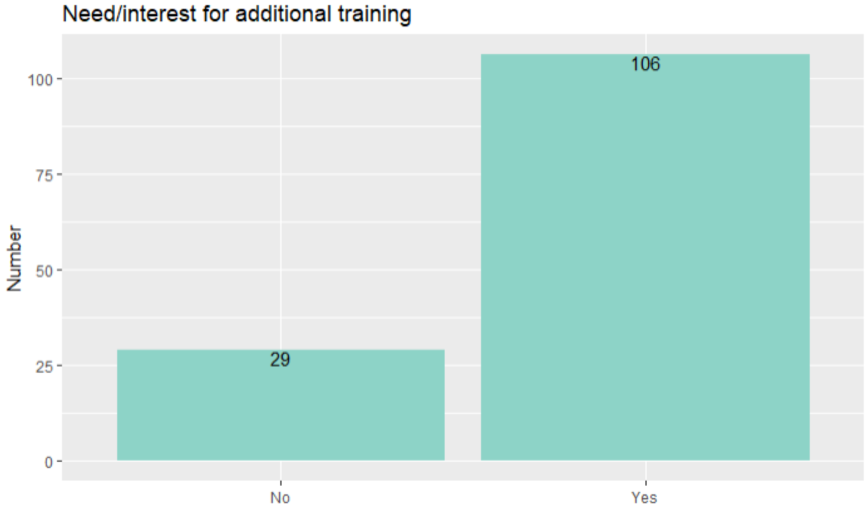

Please indicate on a scale from 1 to 5 how confident you are in working with the following data formats or output (n=116):

	**1**	**2**	**3**	**4**	**5**	**I have no idea what this is**
FASTQ: text-based format for storing raw unaligned sequences and their corresponding quality scores	23%	11%	7%	13%	17%	28%
BAM/SAM (Sequence Alignment/Map): aligned sequences	27%	8%	12%	11%	9%	34%
GFF/GTF and BED: formats for annotation with information and scores	30%	10%	7%	5%	5%	42%
VCF (Variant Call Format): text file for storing gene sequence variations	29%	16%	6%	3%	8%	38%
Data on resistance-conferring mutations present in the genome	28%	16%	12%	9%	9%	26%
Phylogeny inferred from Single Nucleotide Polymorphisms (SNPs)	29%	13%	13%	9%	8%	28%
wgMLST/cgMLST profiles	32%	14%	6%	9%	9%	30%
SNP address nomenclature	34%	12%	9%	6%	6%	32%

1.1.4. Current and foreseen NGS activities

Are you currently using/generating NGS data for any pathogen(s) (n=116)?

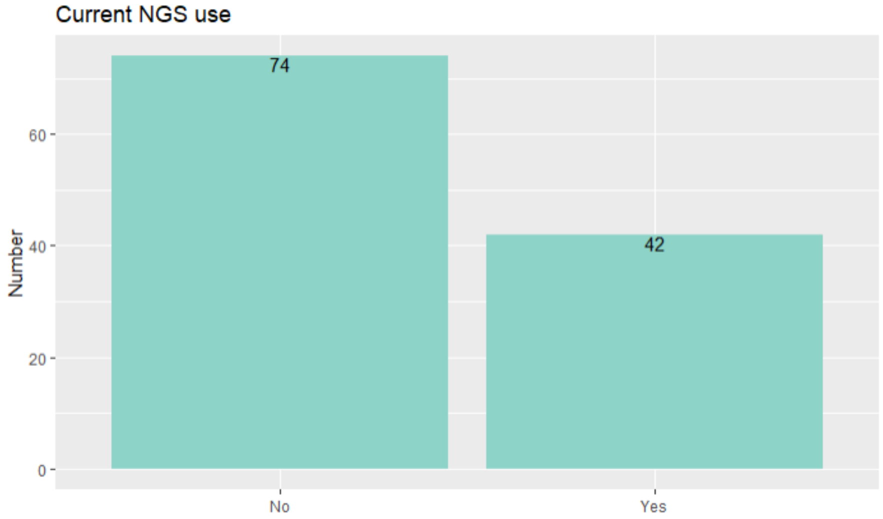

If yes, for which pathogen group(s) and purpose(s)?

	Patient management (diagnosis and/or treatment options)	Outbreak investigations	Control-oriented surveillance	Strategy-oriented surveillance
Respiratory infections (e.g. influenza)	7	8	8	8
Invasive bacterial disease (e.g. N. meningitidis)	5	7	7	6
Vaccine preventable diseases (e.g. measles virus)	3	2	2	3
Food- and waterborne infectious diseases (e.g. Salmonella)	4	16	11	12
Body-fluid related infectious diseases (e.g. HIV, Hepatitis, STI's)	3	0	1	0
Environmental-related diseases (zoonoses, vector-borne) (e.g. malaria)	3	2	1	2
Healthcare-associated infections (e.g. Clostridium difficile, MRSA)	4	5	4	4
Animal diseases	3	2	0	2

If possible, can you specify for which pathogens in particular you are generating/using NGS data?

Pathogens mentioned	Number of times it was mentioned
Salmonella spp.	9
Mycobacterium tuberculosis	8
Escherichia coli (incl. VTEC, STEC)	8
Listeria monocytogenes	7
Influenza	5
Neisseria meningitidis	3
Staphylococcus aureus	3
Legionella pneumophilia	2
Streptococcus pneumoniae	2

HIV	2
Hepatitis C virus	2
ESBL/VRE/CPE	2
Clostridium difficile	1
Enterobacter cloacae	1
Enterococcus faecium	1
Klebsiella pneumoniae	1
Streptococcus pyogenes (GAS)	1
Mycoplasma genitalium (for AB resistance patterns)	1
MDRO colonisation gut	1
Shigella	1
Bordetella pertussis	1
Toxigenic corynebacteria	1

If yes, which strains are subjected to NGS analysis (sampling fraction of the total number of collected strains)?

Subset	Number	Pathogens indicated
All strains	15	STEC, M. tuberculosis, influenza, L. monocytogenes, N. meningitidis
Subset of strains (majority)	8	B. pertussis, influenza, M. bovis, pathogenic E. coli from animals and humans, CPS and CNS from animals
Subset of strains (minority)	19	M. tuberculosis, Legionella, toxigenic corynebacteria, Salmonella spp., influenza, S. pneumoniae, MRSA, Brucella, STEC, Listeria (food), E. coli, hepatitis, L. monocytogenes, Shigella

If yes, is NGS used as a replacement for traditional methods or as a complementary tool?

Implementation	Number	Pathogens indicated
Replacement	23	STEC, M. tuberculosis, C. difficile, B. pertussis, L. monocytogenes, N. meningitidis, influenza, Salmonella spp., L. pneumophilia
Complementary	28	C. difficile, L. monocytogenes, MRSA, M. tuberculosis, STEC, ESBL/VRE/CPE, Salmonella, N. meningitidis, Brucella spp.

Comments :

- ‘We are also investigating the use of MALDI-TOF as a tool to investigate the relatedness between outbreak strains.’
- ‘Like every method in microbiology, NGS is only one method, however modern and performing. It must be applied after definition of a question, work hypothesis and purpose.’
- ‘Because the culture is a good value too!’
- ‘I personally used NGS to help developing another molecular method.’

Are you planning to use/generate NGS data for any (additional) pathogen(s) within the next 3 years (n=116)?

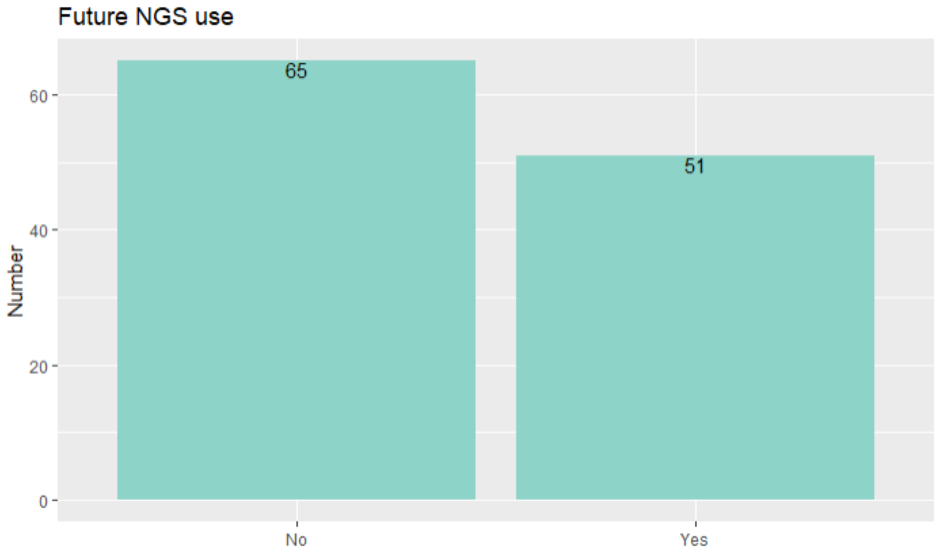

If yes, for which pathogen group(s) and purpose(s)?

	Patient management (diagnosis and/or treatment options)	Outbreak investigations	Control-oriented surveillance	Strategy-oriented surveillance
Respiratory infections (e.g. influenza)	13	8	6	6
Invasive bacterial disease (e.g. N. meningitidis)	11	11	6	5
Vaccine preventable diseases (e.g. measles virus)	4	6	4	6
Food- and waterborne infectious diseases (e.g. Salmonella)	9	15	12	10
Body-fluid related infectious diseases (e.g. HIV, Hepatitis, STI's)	10	2	3	1
Environmental-related diseases (zoonoses, vector-borne) (e.g. malaria)	8	5	4	6
Healthcare-associated infections (e.g. Clostridium difficile, MRSA)	7	11	7	6
Animal diseases	2	5	2	3

If possible, can you specify for which pathogens in particular it is planned to generate/use NGS data within the next 3 years?:

Pathogens mentioned	Number of times it was mentioned
Salmonella spp.	6
Escherichia coli	4
Staphylococcus aureus	5
Clostridium difficile	2
Campylobacter	2
Neisseria gonorrhoeae	2
Bordetella pertussis	2
Klebsiella pneumoniae	2
Enterococcus faecium	2
Listeria monocytogenes	2
RSV	2
hMPV	2
Yersinia spp.	2
Borrelia burghdorferi	1
Tick-borne pathogens	1
Chlamydia trachomatis	1
Mycobacterium tuberculosis	1
Hepatitis B virus	1
Hepatitis C virus	1
Human papilloma virus	1
Cytomegalovirus	1
Epstein-Barr-virus	1
Adenoviruses	1
Varicella Zoster virus	1
Enterovirus	1
Herpes Simplex virus	1
Streptococcus pyogenes (GAS)	1
Enterobacter cloacae	1
Group B streptococci	1
Dermatophytes	1
Measles virus	1
Encephalo tropical viruses	1
VRE	1
Pseudomonas	1
Acinetobacter	1
Citrobacter	1
Candida	1
Norovirus	1
Shigella	1
Legionella pneumophilia	1
Toxigenic corynebacteria	1
Streptococcus pneumoniae	1
Plasmodium spp.	1
Avian influenza H3N1	1

The following comments were provided:

- ‘Unless the cost will decrease, it will be not use in routine in the 3 coming years.’

- ‘We depend on the national reference centres.’
- ‘CNS infections without causal diagnosis after a broad syndromic approach; culture-negative orthopedic infections, culture-negative endocarditis.’
- ‘We would like to explore the use of NGS within the context of orthopaedic infections (implant).’

If not, are there any particular reasons why the implementation of NGS would not be an option?

- ‘Cost and time.’
- ‘Cost and maybe reflexion for NIPT.’
- ‘For pathogens I work on, still only has its place at research level and remains very costly.’
- ‘High cost and lack of experience and reimbursement.’
- ‘High cost, limited additional value for routine clinical lab (for now?).’
- ‘I am not aware what future plan are in our service, although NGS would be interesting for certain pathogens we work with and investigations we do.’
- ‘I don't know the three-year future vision of our lab.’
- ‘I am not working in a lab, but I would be happy to develop recommendations on the management and control of antimicrobial resistance based also on genomics.’
- ‘I only generate the NGS data, the other services that send their DNA determine the planning, I have no idea what their future perspectives are.’
- ‘It costs a lot.’
- ‘Lack of resources and expertise in the team.’
- ‘Mainly financial reasons, no scientific drive to understand transmission within the department.’
- ‘NGS is not that relevant for the pathogen surveillance that I am responsible for, I would if I surveilled pathogens with AMR, high mutation rates, outbreak prone ones with need for identification of the contamination source, high burden of disease (human susceptibilities)...’
- ‘No expertise and not feasible for a first line (private) lab.’
- ‘No good data available, no knowledge, and is it my work as epidemiologist to analyze and show this kind of data?’
- ‘Not really useful actually in the application of procedures in infection control : the general measures of infection control will remain the same.’
- ‘Not sure it'll already be available for every day clinical practice in this interval (but hope I'm wrong).’
- ‘Probably not useful/necessary for detection of a specific pathogen. Maybe implementation of fecal microbiome analysis.’

1.1.5. Key drivers

Which criteria (key drivers) would mainly trigger/advocate the implementation of pathogen genomics in routine public health activities?

Criteria	Mean score all (n=116)	Mean score data providers (n=62)	Mean score data end-users (n=54)
Clinical and/or public health significance	4.28	4.36	4.19
Availability of expertise to generate, analyze and interpret WGS data	4.17	4.31	3.98
Priority with respect to preventing the spread of antimicrobial resistance	4.08	4.11	4.04
Impact on outcomes for patients and populations (translation into actionable results)	3.99	3.91	4.08
Availability of the appropriate infrastructure (sequence technology, high-performance computing, data storage, etc.)	3.99	4.05	3.91
Availability of validated (quality-controlled) WGS workflows (both wet and dry laboratory)	3.90	3.97	3.79
Cost-effectiveness (e.g. replacing multiple tests)	3.88	3.93	3.82

Availability of WGS typing schemes and reference databases (e.g. for antimicrobial resistance)	3.91	3.98	3.81
Possibility to link genomic data from different sources (food-animal-human-environment)	3.86	3.90	3.82
Utility of increased resolution to infer relatedness that would not be obtained via conventional methods	3.85	3.90	3.80
Availability of high-quality/complete/standardized epidemiological and/or clinical data to provide context to the WGS results	3.77	3.91	3.60
Importance of prevention and control programs (e.g. vaccination)	3.73	3.80	3.63
Time-saving compared to conventional testing methods	3.67	3.68	3.65
Local/national/international policy surveillance priorities or obligations	3.57	3.89	3.23
Utility of WGS for diagnostics and/or treatment decisions (individual patient care)	3.34	3.48	3.18

Would you rate one or more of the criteria (drivers) indicated above differently according to the pathogen you have in mind? Would the importance of the criteria depend on the pathogen? If applicable, please specify which criteria, and how its importance would change depending on the pathogen.

- ‘Depends on the evolution in phenotypic typing (e.g. MALDI-TOF for identification & MALDI-AST for fast track resistance pattern identification) - unclear but TAT is much lower...so (?).’
- ‘For mycobacterium it is very important to identify if it is the same pathogen in a specific population.’
- ‘High risk class pathogens are likely to benefit more than lower risk class pathogens.’
- ‘Metagenomics for patients with no identified cause of illness using conventional methods (test sensitivity) and metagenomics for pathogens that today are not diagnosed or unknown.’
- ‘The answers are corresponding to an ideal world. The reality is that (in infectious diseases for public health) the main driver is the pressure exerted by ECDC rather than a real need for public health. It is not because the tool exists that we have to use it. We first have to identify the objectives and evaluate the added value of the new technologies. The first and main drive should be clinical significance: improve quality of care for patient. The second one should be whether the technique could be an added value for prevention and control measures.’
- ‘This would be true for pathogens that can spread via the surroundings, to determine if there is a link or not.’
- ‘For bacteria, NGS will never replace fully classical methods for resistance testing, but would offer important complementary data.’
- ‘The importance of the criteria would depend on the pathogen. Cost-effectiveness (e.g. replacing multiple tests): not particularly true for viruses, but obvious for bacteria.’
- ‘Plasmodium: surveillance of low-level resistance; S. aureus: epidemiology, transmission of virulence and resistance; CPE: spread, evolution of resistance.’
- ‘Priority for resistance in M. tuberculosis strains and in Enterobacteriaceae (CPE), besides typing of HCV & follow-up of resistance in HIV.’

Are there any additional criteria (key drivers) worth considering for the decision of implementing next-generation sequencing (NGS) for public health activities related to a particular pathogen?

- ‘A better collaboration between the veterinary and human side might increase the use of NGS on the veterinary side. An important outbreak in humans of a pathogen/AMR related to food or animals will also trigger the use.’
- ‘Correlation between genomics and infectivity, especially for pathogens that do not grow in conventional cultures.’

- ‘Ethics! Do we need high cost tech to perform surveys? Make it useful and compatible with the budget of the social security.’
- ‘Financing for highly trained staff, infrastructure,...’
- ‘Interpretation of results by taking into account the clinical setting, significance of results in the clinical setting. What to do with results with presence of germs for which pathogenicity has not been established?’
- ‘Local application at the point of problems (Service, Daycare center, Nursing home, farm, milk factory.....).’
- ‘No MLST or other specific typing method available.’
- ‘Outbreak information, how disease spreads, introduction of new pathogens, genetic drift/shift from wild type compared to vaccines strains.’
- ‘Outbreak investigation (and control) is the most interesting use to me.’
- ‘RIZIV reimbursement’
- ‘The perceived utility and feasibility appears to be a key driver for the implementation of this technology potentially of more importance than any of the key drivers listed in the previous questions.’
- ‘The importance of science is in daily practice rather than just preventive actions.’

1.1.6. Expected outputs

The output from WGS needs to be in a format that is useful for its end-users. In order to facilitate the translation of complex genomic data into actionable results, which kind of data formats should be delivered following NGS analyses?

Output format	Number	Pathogens and applications indicated
Whole-genome phylogenetic tree (identification of clusters)	46	M. tuberculosis, bacterial foodborne pathogens, MDRO, L. monocytogenes, N. meningitides, enteroviruses, measles virus, influenza, norovirus.<br>Outbreak investigations
Whole-genome phylogenetic tree combined with epidemiological links or exposures	50	M. tuberculosis, bacterial foodborne pathogens, MDRO<br>Outbreak investigations, epidemics
Table on clustered cases	33	M. tuberculosis, bacterial foodborne pathogens, L. monocytogenes, STEC, Salmonella spp.<br>Detection of outbreaks
Line listings of resistance/virulence-conferring mutations (virulome/resistome)	46	M. tuberculosis, bacterial foodborne pathogens, MDRO, Salmonella, Shigella, Hepatitis C virus, HIV, Enterobacteriaceae, M. genitalum, N. gonorroeae<br>Diagnosis
Line listings of isolate pathovar name - antibiogramme profile	33	Encephalitis germs (e.g. TBEV in CSF, Leptospira, flaviviridae), AMR<br>Diagnosis

Other:

- ‘Geographical link.’
- ‘Translation into a readable format.’
- ‘Depends on the pathogen and information of interest.’

What would be the desired turn-around time?

Turn-around time	Number	Application
< one week	64	Outbreak investigations, diagnosis (identification, pathotype, resistotype)
< one month	22	Surveillance, clustering, outbreak investigations
< three months	4	Surveillance
< one year	0	

Other:

- ‘Preferably in real time using newer technology like minion.’

- ‘< one day and if possible under a couple of hours would be preferable!’
- ‘In case of outbreaks speed is relevant.’
- ‘< 3 hours’.
- ‘For diagnosis it should be in real time.’
- ‘For diagnosis, then in real time.’
- ‘<10 days.’
- ‘Depends on the set up: acute outbreak (e.g; Legionellosis) versus progressive evolving problem (HIV-epidemic).’
- ‘When linked with epidemiologic inquiry in the field, I do not urgently need the data. They will be analyzed together with contact structure.’

1.1.7. Diagnostic hierarchy

Which WGS provision model seems most appropriate in the Belgian context, in your opinion?

1. Centralization of sequencing and bioinformatics at one central sequencing center
2. Centralization of sequencing and bioinformatics at National Reference Centers (which are organized per pathogen or group of pathogens)
3. Decentralized point-of-care sequencing (at frontline laboratories), but centralization of bioinformatics (mixed model)
4. Decentralization of sequencing and bioinformatics, but mandatory submission of isolates to a national repository
5. Decentralization of sequencing and bioinformatics, but mandatory submission of raw sequence data to a national repository
6. Decentralization of sequencing and bioinformatics, but mandatory submission of bioinformatics output to a national repository

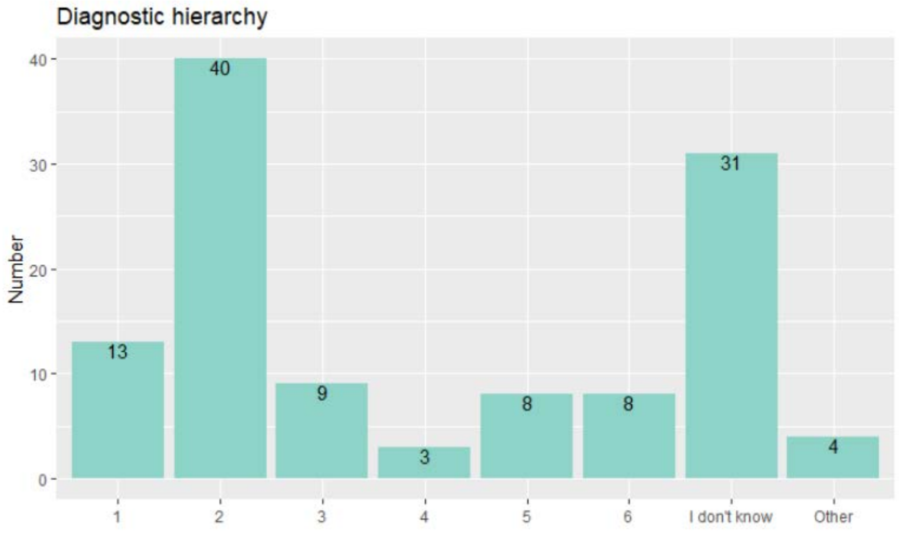

Other?

- ‘Centralization of sequencing and bioinformatics at one or few central sequencing centers.’
- ‘Decentralized or centralized sequencing, centralized bioinformatics in 2-5 expert centers.’
- ‘Depending on what is most cost-effective. But should be overall coordinated and controlled by the federal public health authority.’
- ‘It depends how fast this technique will be implemented in routine laboratories. In any scenario it will be important that sequence data are brought together in one databank for surveillance purposes (trends, outbreaks, resistance, etc..).’

1.1.8. Data sharing

What are according to you the main obstacles for sharing pathogen sequence data and associated metadata (n=116)?

Issues	Major obstacle	Minor obstacle	No obstacle	Don't know
Practical barriers: lack of data standardization, poor data quality, missing meta-data, etc.	68	30	3	15
Ethical issues and concerns: accompanying patient data might give rise to privacy issues (personal data protection)	44	44	13	15
Political sensitivities: potential misuse of data, national security and safety, economic risks, etc.	41	50	8	17
Priority to publication: concerns over misuse and inability to publish	31	47	16	22

Are there any additional obstacles for sharing pathogen sequence data and associated metadata not mentioned above, and/or would you like to make some comments?

- 'Common as well as individual effort should be recognized.'
- 'Ethical issues related to patients, actions of HCW but also transmission via HCW...'
- 'It is really a pity priority to publication is an obstacle in the scientific world as it functions know.'
- 'In this stage, a lot of work is done in het framework of research. Data are only shared after publication of articles.'
- 'Mistrust between institutes and or persons; lack of communication about the purpose of the data sharing.'
- 'No central BE or EU organization.'
- 'No central database, no clear guidelines on how and what to share.'
- 'The bureaucracy involved in the transmission of data. E.g. data transmission for NGS in myeloid tumor organized by Sciensano: this is a complete disaster! Input at 3 different sites, the same data have to be supplied twice in different sites!'
- 'The structure of public health in Belgium will not help sharing data.'
- 'The required technical infrastructure: hosting one data repository for all European WGS data itself is a major technical bottleneck that will take a lot of money to implement.'
- 'Need to differentiate between "public access" and "access between partners involved in outbreaks" e.g. EPIS: you can share information and exchange in a closed environment with confidential information (no public access).'

If you have any comments on the survey or the project, please leave a comment below.

- 'I am sure that the WGS has an interest in outbreak investigations in hospitals. However, the lack of training and knowledge of health practitioners and how to use WGS in these investigations is a barrier to the development of these new technologies. From when do they have to use NGS for a multi-resistant bacteria? On how many contaminated bacteria/patients? How many samples should they analyze and how to interpret these results? These are all questions that will have to be developed in the future, in consultation with the hospital and laboratory sector.'
- 'Just one comment on this sentence: "Data on resistance-conferring mutations present in the genome (e.g. ResFinder)". Historically ResFinder has been used to detect the presence of AMR genes (not mutations!). The newest versions also report the specific point mutations because PointFinder has been integrated into ResFinder. But this might possibly be confusing for people who are only familiar with the old ResFinder. And most people who I know that use ResFinder are more interested in the genes than the point mutations.'
- 'This questionnaire is human-orientated and not enough animal orientated.'
