# Supplementary material for: Perceived utility and feasibility of pathogen genomics for public health practice: a survey among public health professionals working in the field of infectious diseases, Belgium, 2019
Source: BMC Public Health. 2020 Aug 31;20:1318. doi: 10.1186/s12889-020-09428-4 (PMC7456758; doi:10.1186/s12889-020-09428-4)
Supplement: Supplementary file 4 — Additional file 4. “Thematic analysis of open questions”. Description of data: “A full list of identified themes and the coded text”. [file 12889_2020_9428_MOESM4_ESM.pdf]

<u>**Thematic analysis of open questions**</u>

Code	Coded text
Feasibility: capacity building	Appropriate training of personnel for execution and interpretation.
Feasibility: capacity building	Extremely important technology in which it is needed to invest so that sufficient capacity is built.
Feasibility: capacity building	I am sure that the WGS has an interest in outbreak investigations in hospitals. However, the lack of training and knowledge of health practitioners and how to use WGS in these investigations is a barrier to the development of these new technologies. From when do they have to use NGS for a multi-resistant bacteria? On how many contaminated bacteria/patients? How many samples should they analyze and how to interpret these results? These are all questions that will have to be developed in the future, in consultation with the hospital and laboratory sector.
Feasibility: capacity building	I'm a nurse infection prevention and this subject was never an issue in trainings or symposium.
Feasibility: capacity building	Lack of experience.
Feasibility: capacity building	Lack of resources and expertise in the team.
Feasibility: capacity building	Lack of training adapted to public health needs.
Feasibility: capacity building	Is it my work as epidemiologist to analyze and show this kind of data?
Feasibility: capacity building	No expertise and not feasible for a first line (private) lab.
Feasibility: capacity building	No interest for my work/tasks.
Feasibility: capacity building	No knowledge.
Feasibility: capacity building	No scientific drive to understand transmission within the department.
Feasibility: capacity building	Not a priority in my profession.
Feasibility: capacity building	Not applicable for a clinician.
Feasibility: capacity building	Not my priority, others are already more qualified.
Feasibility: capacity building	The exact topic of this survey, i.e. the perceived utility and feasibility of pathogen genomics by public health practitioners , which is the biggest bottleneck of all. All the other concerns listed above can be tackled given the drive within the field to solve them in the first place.
Feasibility: capacity building	The perceived utility and feasibility appears to be a key driver for the implementation of this technology potentially of more importance than any of the key drivers listed in the previous questions
Feasibility: capacity building	Training
Feasibility: contextual data	Data collection is already limited so newer technologies will not automatically improve this process but be redundant if the basics are not met.
Feasibility: contextual data	Harmonization of epidemiological data. Most of the epidemiological data is very 'messy' or inconsistent, which makes systematic integration and surveillance unfeasible.
Feasibility: contextual data	No good data available.
Feasibility: costs	Ethics ! Do we need high cost tech to perform surveys ? Make it useful and compatible with the budget of the social security.
Feasibility: costs	Financing ;-) for highly trained staff, infrastructure,...
Feasibility: costs	For pathogens I work on, still only has its place at research level and remains very costly.
Feasibility: costs	High cost.
Feasibility: costs	High cost and lack of experience and reimbursement.
Feasibility: costs	It costs a lot.
Feasibility: costs	Mainly financial reasons.
Feasibility: costs	RIZIV reimbursement.

Feasibility: costs	The unit price for each analysis, although decreasing over time will be a limit and not all the labs could train and keep specialists in data analysis at work => the time limit for this vision (5years) might be too short... but with the automatisation of the analysis process... it will become affordable.
Feasibility: costs	To understand and see cost/benefit with the all pictures (often the view is fragmented analysis by analysis).
Feasibility: costs	Unless the cost will decrease, it will be not use in routine in the 3 coming years.
Feasibility: costs	Who will sponsor all this?
Feasibility: data sharing	Common as well as individual effort is should be recognized.
Feasibility: data sharing	In this stage, a lot of work is done in het framework of research. Data are only shared after publication of articles.
Feasibility: data sharing	International compatibilities.
Feasibility: data sharing	It is really a pity priority to publication is an obstacle in the scientific world as it functions know.
Feasibility: data sharing	Mistrust between institutes and or persons; lack of communication about the purpose of the data sharing.
Feasibility: data sharing	No central BE or EU organization.
Feasibility: data sharing	No central database, no clear guidelines on how and what to share.
Feasibility: data sharing	Open access.
Feasibility: data sharing	Standardization and facilities for data sharing need to be improved.
Feasibility: data sharing	The bureaucracy involved transmission of data: the example here is the data transmission for NGS in myeloid tumor organized by Sciensano: this is a complete disaster!! Input at 3 different sites, the same data have to be supplied twice in different sites!
Feasibility: data sharing	The structure of public health in Belgium will not help sharing data.
Feasibility: data sharing	Yes the required technical infrastructure. Hosting one data repository for all European WGS data itself is a major technical bottleneck that will take a lot of money to implement.
Feasibility: data sharing	You may differentiate "public access" and "access between partners involved in outbreaks" e.g. epis: you can share information and exchange in an closed environment with confidential information (no public access)
Feasibility: ethics	Ethical issues related to patients, actions of HCW but also diseases via HCW....
Feasibility: ethics	Ethics ! Do we need high cost tech to perform surveys ? Make it useful and compatible with the budget of the social security.
Feasibility: ethics	Healthcare workers integrity concerns.
Feasibility: ethics	In the HIV field, the phylogenetic analyses of virus permit to have an hindsight in paths of transmission. It is a very tricky topic in ethical and potentially legal aspects.
Feasibility: multi-disciplinary working	Interpretation across sectors
Feasibility: multi-disciplinary working	Multidisciplinary knowledge of personal working on this topic.
Feasibility: multi-disciplinary working	Communication within Belgium and across borders.
Feasibility: timeliness	Depends on the set up: acute outbreak (e.g.; Legionellosis) versus progressive evolving problem (HIV-epidemic).
Feasibility: timeliness	When linked with epidemiologic inquiry in the field, I do not urgently need the data. They will be analyzed together with contact structure.
Feasibility: timeliness	3 hours
Feasibility: timeliness	Between 1 and 2 weeks, less in case of urgency (outbreaks with high mortality rate and/or morbidity rate).

Feasibility: timeliness	Depends on the evolution in phenotyping typing (e.g. MALDITOF for identification & MALDI-AST for fast track resistance pattern identification) - unclear but TAT is much lower...so (?)
Feasibility: timeliness	It really depends of the objective. If for diagnosis, then in real time.
Feasibility: timeliness	Rapidity to obtain the sequences (difficulty to have it in real time) , possibility to mix virus and bacteria to increase the rapidity to obtain results.
Feasibility: wet and dry lab	Culture is a good value too.
Feasibility: wet and dry lab	I personally used NGS to help developing another molecular method.
Feasibility: wet and dry lab	Reliability of generated data; high interlab variation.
Feasibility: wet and dry lab	Storage of data.
Feasibility: wet and dry lab	The "kitome" problem, contaminated reagents kits, which becomes more and more clear while performing WGS/NGS in microbiology.
Feasibility: wet and dry lab	We are also investigating the use of MALDI-TOF as a tool to investigate the relatedness between outbreak strains.
One-Health context	This questionnaire is human-orientated and not enough animal orientated.
One-Health context	A better collaboration between the veterinary and human side might increase the use of NGS on the veterinary side. An important outbreak in humans of a pathogen/AMR related to food or animals will also trigger the use.
One-Health context	Monitoring the emergence and spread of zoonotic pathogens has been impacted negatively, by the introduction of WGS at the human site only: I don't see this changing in the next 5 years unfortunately
Routine implementation	A 5 year delay is probably too short, it will be not yet done in routine, it will be more for research.
Routine implementation	For pathogens I work on, still only has its place at research level and remains very costly.
Routine implementation	How to interpret the result at clinical level.
Routine implementation	Implementation of the NGS results in the legislation and acceptance of the use of NGS for routine in enforcement laboratories by the competent authorities. For this they need to have a basic understanding (education) it order to understand and see cost/benefit with the all pictures (often the view is fragmented analysis by analysis).
Routine implementation	Limited additional value for routine clinical lab (for now?)
Routine implementation	Local application at the point of problems (Service, Daycare center, Nursing home, farm, milk factory.....)
Routine implementation	Metagenomics in clinical practice will not be implemented in little to moderate hospitals within the first 5y I'm afraid. Sciensano can offer solutions.
Routine implementation	Not sure it'll already be available for every day clinical practice in this interval (but hope I'm wrong)
Routine implementation	Unless the cost will decrease, it will be not use in routine in the 3 coming years.
Routine implementation: organization	Centralization of sequencing and bioinformatic at one or few central sequencing centers.
Routine implementation: organization	Decentralized or centralized sequencing, centralized bioinformatics in 2-5 expert centers.
Routine implementation: organization	Depending on what is most cost-effective. But should be overall coordinated and controlled by the federal public health authority.
Routine implementation: organization	It depends how fast this technique will be implemented in routine laboratories. In any scenario it will be important that sequence data are brought together in one databank for surveillance purposes (trends, outbreaks, resistance, etc..)

Routine implementation: organization	The only real and the major concern is the fear that some actors in the field will try to abuse their power and influence to monopolize this new technology to only university hospitals or only to public health authorities like Sciensano. To really be valuable to patient management and public health it is absolutely required to offer free access to all laboratories to this new technology.
Routine implementation: organization	We depend on the national reference centers.
Routine implementation: translation into action	Correlation between genomics and infectivity, especially for pathogens that do not grow in conventional cultures.
Routine implementation: translation into action	Does identification prove that there are risks, risk is linked to quantity, establishing this limits will take time. The number of bacteria, viruses etc. is infinite and we don't know yet the role (microbiome) of all of them, individual and together.
Routine implementation: translation into action	Interpretation of results by taking into account the clinical setting, significance of results in the clinical setting. What to do with results with presence of germs for which pathogenicity has not been established?
Routine implementation: translation into action	Like every method in microbiology, NGS is only one method, however modern and performing. It must be applied after definition of a question, work hypothesis and purpose.
Routine implementation: translation into action	Not really useful actually in the application of procedures in infection control: the general measures of infection control will remain the same.
Routine implementation: translation into action	The answers are corresponding to an ideal world. The reality is that (in infectious diseases for public health) the main driver is the stress done by ECDC than a real need for public health. The tool exists so we have to use before to have identify the objectives and evaluate the added value of the new technologies. The first and main drive should be clinical significance, improve quality of care for patient. The second one if the technique could be an added value for prevention and control measures.
Routine implementation: translation into action	The importance of science is in daily practice rather than just preventive actions.
Routine implementation: translation into action	Translation into a readable information.
Utility (applications)	For mycobacterium it’s very important to identify if it’s the same pathogen in a specific population.
Utility (applications)	CNS infections without causal diagnosis after a broad syndromic approach; culture-negative orthopedic infections, culture-negative endocarditis.
Utility (applications)	Air quality
Utility (applications)	Discovery of causal relation between a pathogen and a clinical disease (e.g. cancer)
Utility (applications)	Drinking water quality
Utility (applications)	Early diagnostics of diseases due to slowly growing pathogens (Tuberculosis)
Utility (applications)	For pathogens that can spread via the surroundings, to determine if there is a link or not.
Utility (applications)	General characterization of pathogens.
Utility (applications)	High risk class pathogens are likely to benefit more than lower risk class pathogens
Utility (applications)	Home environmental quality (molds, yeasts)
Utility (applications)	Identification and characterization of new strains
Utility (applications)	Identification of new clones
Utility (applications)	Influence on appropriate antibiotic use or no use and diminishing the overall rate of resistance
Utility (applications)	Insight in dysbiosis
Utility (applications)	International tracking
Utility (applications)	Maybe implementation of fecal microbiome analysis.

Utility (applications)	Metagenomics for patients with no identified cause of illness using conventional methods (test sensitivity) and metagenomics for pathogens that today are not diagnose or unknown.
Utility (applications)	Monitoring of antiviral resistance
Utility (applications)	Monitoring of homology with vaccine strains (Influenza).
Utility (applications)	Monitoring trends in resistance of HIV - already widely used for years at the moment of diagnosis of HIV infection
Utility (applications)	MTB
Utility (applications)	Control on antibiotic use in livestock breeding
Utility (applications)	CPE: spread, evolution of resistance
Utility (applications)	Early identification/diagnosis of emerging diseases
Utility (applications)	Genetic drift/shift from wild type compared to vaccines strains
Utility (applications)	Introduction of new pathogens
Utility (applications)	No MLST or specific typing method
Utility (applications)	Phylogenetic analysis to better understand the spread of the HIV infection
Utility (applications)	S. aureus: epidemiology, transmission of virulence and resistance
Utility (applications)	Outbreak information, how disease spreads
Utility (applications)	Outbreak investigation (and control) is the most interesting use to me
Utility (applications)	Pathogenicity (identification of virulence factors)
Utility (applications)	Pathogens discovery (molecular identification and characterization of new putative pathogens)
Utility (applications)	Pathogens with AMR, high mutation rates, outbreak prone ones with need for identification of the contamination source, high burden of disease (human susceptibilities)...
Utility (applications)	Phage therapy
Utility (applications)	Phylogenetic studies in an epidemiological context
Utility (applications)	Plasmodium: surveillance of low-level resistance
Utility (applications)	Priority for resistance in M. tuberculosis strains and in Enterobacteriaceae (CPE)
Utility (applications)	Probably not useful/necessary for detection of a specific pathogen.
Utility (applications)	The importance of the criteria would depend on the pathogen. Ex: Cost-effectiveness (e.g. replacing multiple tests): not particularly true for viruses, but obvious for bacteria
Utility (applications)	To identify the possible origin of a bacteria or virus whether it was imported or circulated within Belgium
Utility (applications)	Typing of HCV & follow-up of resistance in HIV
Utility (applications)	Vaccine development
Utility (applications)	We would like to explore the use of NGS within the context of orthopedic infections (implant).
Utility (applications)	For bacteria, NGS will never replace fully classical methods for resistance testing, but would offer important complementary data.
