# Supplementary figures and images for: Perceived utility and feasibility of pathogen genomics for public health practice: a survey among public health professionals working in the field of infectious diseases, Belgium, 2019
Source: BMC Public Health. 2020 Aug 31;20:1318. doi: 10.1186/s12889-020-09428-4 (PMC7456758; doi:10.1186/s12889-020-09428-4)

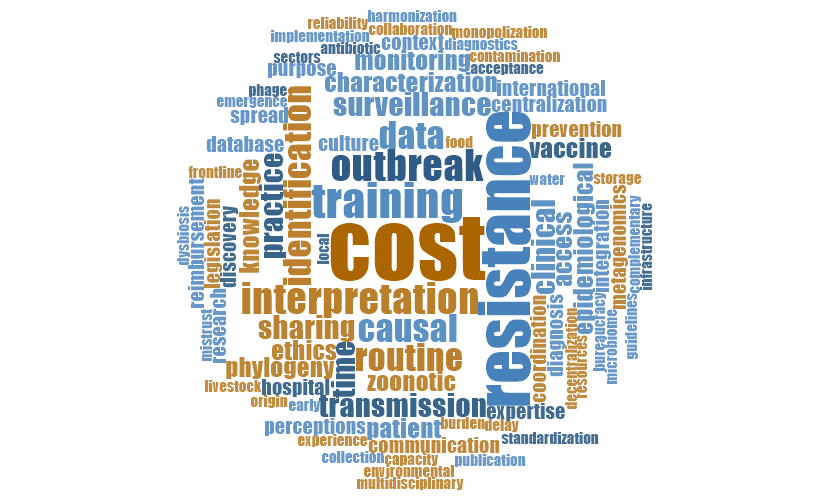

Supplement: Supplementary file 5 — Additional file 5. “Word cloud based on open questions”. Description of data: “Word cloud constructed based on the free text responses visualizing the word frequency”. [file 12889_2020_9428_MOESM5_ESM.png]
